# The Sec61 translocon is a therapeutic vulnerability in multiple myeloma

**DOI:** 10.15252/emmm.202114740

**Published:** 2022-01-11

**Authors:** Antoine Domenger, Caroline Choisy, Ludivine Baron, Véronique Mayau, Emeline Perthame, Ludovic Deriano, Bertrand Arnulf, Jean‐Christophe Bories, Gilles Dadaglio, Caroline Demangel

**Affiliations:** ^1^ Unité d’Immunobiologie de l’Infection Institut Pasteur INSERM U1224 Université de Paris Paris France; ^2^ Sorbonne Paris Cité Université de Paris Paris France; ^3^ INSERM U976 Institut de Recherche Saint Louis Université de Paris Paris France; ^4^ Bioinformatics and Biostatistics Hub Institut Pasteur Université de Paris Paris France; ^5^ Unité d’Intégrité du Génome Immunité et Cancer Equipe Labellisée Ligue Contre Le Cancer Institut Pasteur INSERM U1223 Université de Paris Paris France; ^6^ APHP Department of Immuno‐Hematology Hôpital Saint Louis Paris France

**Keywords:** multiple myeloma, proteostatic stress, Sec61 translocon, Cancer, Haematology

## Abstract

Multiple myeloma (MM) is an incurable malignancy characterized by the uncontrolled expansion of plasma cells in the bone marrow. While proteasome inhibitors like bortezomib efficiently halt MM progression, drug resistance inevitably develop, and novel therapeutic approaches are needed. Here, we used a recently discovered Sec61 inhibitor, mycolactone, to assess the interest of disrupting MM proteostasis via protein translocation blockade. In human MM cell lines, mycolactone caused rapid defects in secretion of immunoglobulins and expression of pro‐survival interleukin (IL)‐6 receptor and CD40, whose activation stimulates IL‐6 production. Mycolactone also triggered pro‐apoptotic endoplasmic reticulum stress responses synergizing with bortezomib for induction of MM cell death and overriding acquired resistance to the proteasome inhibitor. Notably, the mycolactone–bortezomib combination rapidly killed patient‐derived MM cells *ex vivo*, but not normal mononuclear cells. In immunodeficient mice engrafted with MM cells, it demonstrated superior therapeutic efficacy over single drug treatments, without inducing toxic side effects. Collectively, these findings establish Sec61 blockers as novel anti‐MM agents and reveal the interest of targeting both the translocon and the proteasome in proteostasis‐addicted tumors.

## Graphical abstract

In this work, a pathogen‐derived inhibitor named mycolactone was used to demonstrate that the Sec61 translocon is a therapeutic target in Multiple Myeloma (MM).

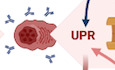

**The paper explained**

**Problem**

Multiple myeloma is an incurable blood cancer. While the majority of patients initially respond to combination chemotherapies, all eventually develop drug resistance or toxicity and their mean survival rate is only 6 years post‐diagnosis.

**Results**

Human cells interact with their environment via proteins that are either expressed on their surface or secreted into the extracellular environment. The process of delivering these proteins to the membrane wall or outside – the secretion pathway – uses a dedicated distribution network whose gateway is the Sec61 translocon. We recently found that mycolactone, a toxin produced by a bacterial pathogen, operates by blocking Sec61. Using mycolactone we demonstrate in the present study that blocking Sec61 is much more toxic to multiple myeloma cells than to normal cells *in vitro* and *in vivo*. Moreover, we show that mycolactone potentiates the activity of drugs forming the backbone of current multiple myeloma chemotherapies.

**Impact**

Our findings identify Sec61 as a therapeutic vulnerability in multiple myeloma, and potentially all other cancers needing an active Sec61 translocon to survive. They suggest that treating MM patients with agents blocking Sec61 will augment the efficacy of – and overcome resistance to – current chemotherapies.

## Introduction

Multiple myeloma (MM) is a malignant disorder characterized by the uncontrolled expansion of clonal plasma cells in the bone marrow, eventually leading to organ dysfunction and death (Laubach *et al*, 2011). The global burden of MM has increased by 126% over the past 30 years, with highest incidences in developed countries (Cowan *et al*, 2018). The introduction of proteasome inhibitors (PIs) like bortezomib (BZ) in MM chemotherapies has revolutionized MM management, and combinations of BZ with immunomodulators (IMiDs) and dexamethasone have become the gold standard for MM treatment (Kouroukis *et al*, 2014). However, BZ can induce severe side effects such as peripheral neuropathy, requiring discontinuation of therapy (Lawasut *et al*, 2012). Moreover, many patients develop resistance to BZ whose molecular basis remains poorly understood, and less than half of them survive beyond 5 years post‐diagnosis. Second generation PIs were recently developed to treat patients who are resistant or intolerant to BZ (Gandolfi *et al*, 2017), but these drugs induce other types of side effects and do not prevent relapse. Therefore, despite significant therapeutic advances, MM remains an incurable disease and the identification of new therapeutic targets is critically needed.

The clinical efficacy of BZ is primarily attributed to its ability to induce the accumulation of misfolded proteins including immunoglobulins in the cytoplasm of MM cells, leading to lethal proteotoxic stress (Gandolfi *et al*, 2017). BZ also alters the survival and proliferation of MM cells in several other ways, such as inhibition of NF‐κB oncogenic signaling, suppression of pro‐adhesive cross‐talks with bone marrow stromal cells, and prevention of growth stimulation by cytokines like interleukin (IL)‐6 (Mahindra *et al*, 2010; Rosean *et al*, 2014). Among the different strategies that are currently explored to complement or replace PIs in MM chemotherapies are novel means to disrupt the homeostatic regulation of the secretory apparatus via proteolytic routes or endoplasmic reticulum (ER)‐associated protein degradation (ERAD) components other than the proteasome (Auner & Cenci, 2015). Despite its central importance in MM cell biology, the interest of targeting immunoglobulin transport into the ER has not yet been explored due to the lack of suitable inhibitor.

We reported recently that mycolactone, a diffusible lipid toxin secreted by the human pathogen *Mycobacterium ulcerans*, operates by inhibiting the mammalian translocon (Sec61) (Baron *et al*, 2016; Demangel & High, 2018). By targeting the central pore‐forming subunit of Sec61 (Sec61α), mycolactone prevents the import of newly synthesized secreted and transmembrane proteins into the ER, leading to their cytosolic degradation by the proteasome (Hall *et al*, 2014; McKenna *et al*, 2016, 2017). Contrary to the Sec61 inhibitor cotransin (Mackinnon *et al*, 2014), mycolactone is not substrate selective and blocks the translocation of the vast majority of Sec61 clients (Baron *et al*, 2016; Morel *et al*, 2018). The only substrates resisting its inhibitory action are transmembrane proteins belonging to the rare Type III subgroup (McKenna *et al*, 2016, 2017; Demangel & High, 2018; Morel *et al*, 2018). Within 1 h of treatment, mycolactone‐treated cells become defective for production of most secreted proteins and membrane‐anchored receptors. If sustained, mycolactone treatment triggers proteotoxic stress responses in cytosol and ER, ultimately inducing apoptosis (Morel *et al*, 2018; Ogbechi *et al*, 2018). Notably, a point mutation (R66G) in the Sec61α amino acid sequences preventing mycolactone binding without affecting the translocon functionality was sufficient to prevent stress responses and associated cytotoxicity, demonstrating that Sec61 inhibition by mycolactone is the molecular mechanism driving cell death (Baron *et al*, 2016).

Based on these findings, we hypothesized that Sec61 blockade may suppress survival and growth of MM cells in two ways: by preventing the expression of membrane receptors that are key to MM cell division and dissemination, and by generating lethal proteotoxic stress. The present study uses mycolactone as a prototypical Sec61 blocker to establish proof of concept, and evaluates the translational potential of Sec61 inhibitors in the treatment of MM.

## Results

### Sec61 blockade by mycolactone alters the biology and viability of MM cell lines

**Figure 1:**
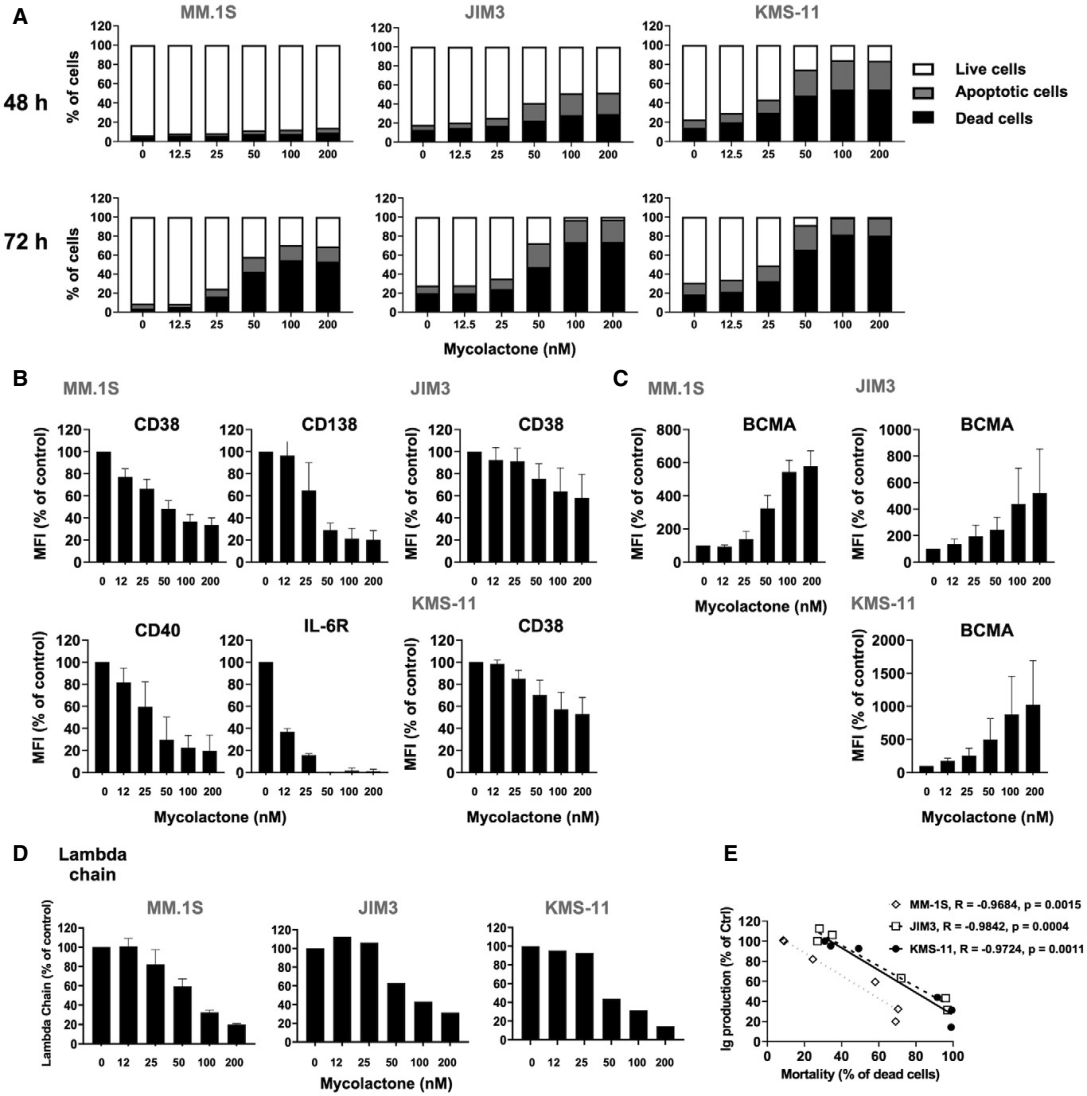
Sec61 blockade by mycolactone strongly alters the biology and viability of MM cell lines AEffect of mycolactone on MM.1S, JIM3, and KMS‐11 cell viability after 48 and 72 h of treatment, as measured by exposure of Annexin V and incorporation of PI. Data are Mean % of live, apoptotic, and dead cells from technical duplicates, gated as in Appendix Fig S1, relative to total cells.B, CInhibitory effect of mycolactone on cell expression of CD38 (Type II) and CD138, IL‐6R, and CD40 (Type I) transmembrane proteins (B). Stimulatory effect of mycolactone on cell expression of BCMA, a Type III transmembrane protein (C). Mean fluorescence intensities (MFIs) were measured in live cells 48 h after addition of mycolactone. Data are Mean MFIs ± SD from technical duplicates of two independent experiments, relative to vehicle‐treated controls (Ctrl).DInhibitory effect of mycolactone on immunoglobulin (lambda chain) secretion, as quantified by ELISA in culture supernatants after 24 h of treatment. Data are Mean concentrations ± SD of secreted lambda chain (µg/ml) from technical duplicates of two independent experiments (MM.1S) or Mean of secreted lambda chain concentrations (µg/ml) from technical duplicates (JIM3 and KMS‐11), relative to vehicle‐treated controls.ECorrelation between immunoglobulin lambda chain (Ig) secretion after 24 h (D) and MM cell line mortality after 72 h (A), in the three cell lines treated with 50 nM mycolactone. Slopes (R) and statistical significance (*P* values) are indicated. Data information: In A (all cell lines) and D (JIM3 and KMS‐11), shown data are representative of two independent experiments with similar results.

To assess the effect of mycolactone on MM cell viability, three human cell lines (MM.1S, JIM3, and KMS‐11) were treated with increasing concentrations of mycolactone for 24–72 h, and the induction of cell apoptosis was monitored by phosphatidylserine exposure (Annexin V staining) and loss of membrane integrity (PI staining) (Appendix Fig S1). While the three cell lines differed in sensitivity to mycolactone, MM.1S being the most resistant, a dose‐ and time‐dependent induction of apoptotic cell death was consistently observed after 48 h of treatment (Fig 1A). In all cell lines, initiation of apoptosis was preceded by a decrease in surface expression of the MM cell marker CD38 (Van De Donk *et al*, 2018) (Fig 1B). The plasma cell marker CD138 supports MM cell survival in the bone marrow by promoting growth factor signaling (Akhmetzyanova *et al*, 2020). While not detected on JIM3 and KMS‐11, CD138 expression by MM.1S was also dose dependently decreased by mycolactone after 24 h (Fig 1B). IL‐6 receptor (IL‐6R) and CD40 are two other MM cell markers whose signaling play a crucial role in MM development and dissemination (Tai *et al*, 2005; Rosean *et al*, 2014). When expressed by the MM cell lines, these receptors were also rapidly and potently downregulated by mycolactone treatment (Fig 1B).

In contrast to CD38, CD138, IL‐6R, and CD40, all Type I or II transmembrane proteins efficiently blocked in translocation by mycolactone; Type III transmembrane proteins are Sec61 substrates resisting mycolactone inhibition (Baron *et al*, 2016; McKenna *et al*, 2016, 2017; Morel *et al*, 2018). B‐cell maturation antigen (BCMA) is a Type III protein that is typically expressed by MM cells and the target of next‐generation immunotherapies (Cho *et al*, 2020). In all cell lines, mycolactone dose dependently increased cell surface expression of BCMA (Fig 1C). On the opposite, secreted proteins are Sec61 substrates that are efficiently blocked in translocation by mycolactone (Baron *et al*, 2016; McKenna *et al*, 2016, 2017; Morel *et al*, 2018). Figure 1D shows that Sec61 inhibition efficiently decreased MM cell line secretion of immunoglobulin light chains after only 24 h of treatment, and this reduction closely correlated with the onset of cell death after 72 h (Fig 1E). In conclusion, Sec61 blockade by mycolactone induces programmed cell death in MM preceded by phenotypic defects in expression of Type I/II membrane receptors and secretion of immunoglobulins.

### Mycolactone synergizes with proteasome inhibitors for induction of MM cell apoptosis

**Figure 2:**
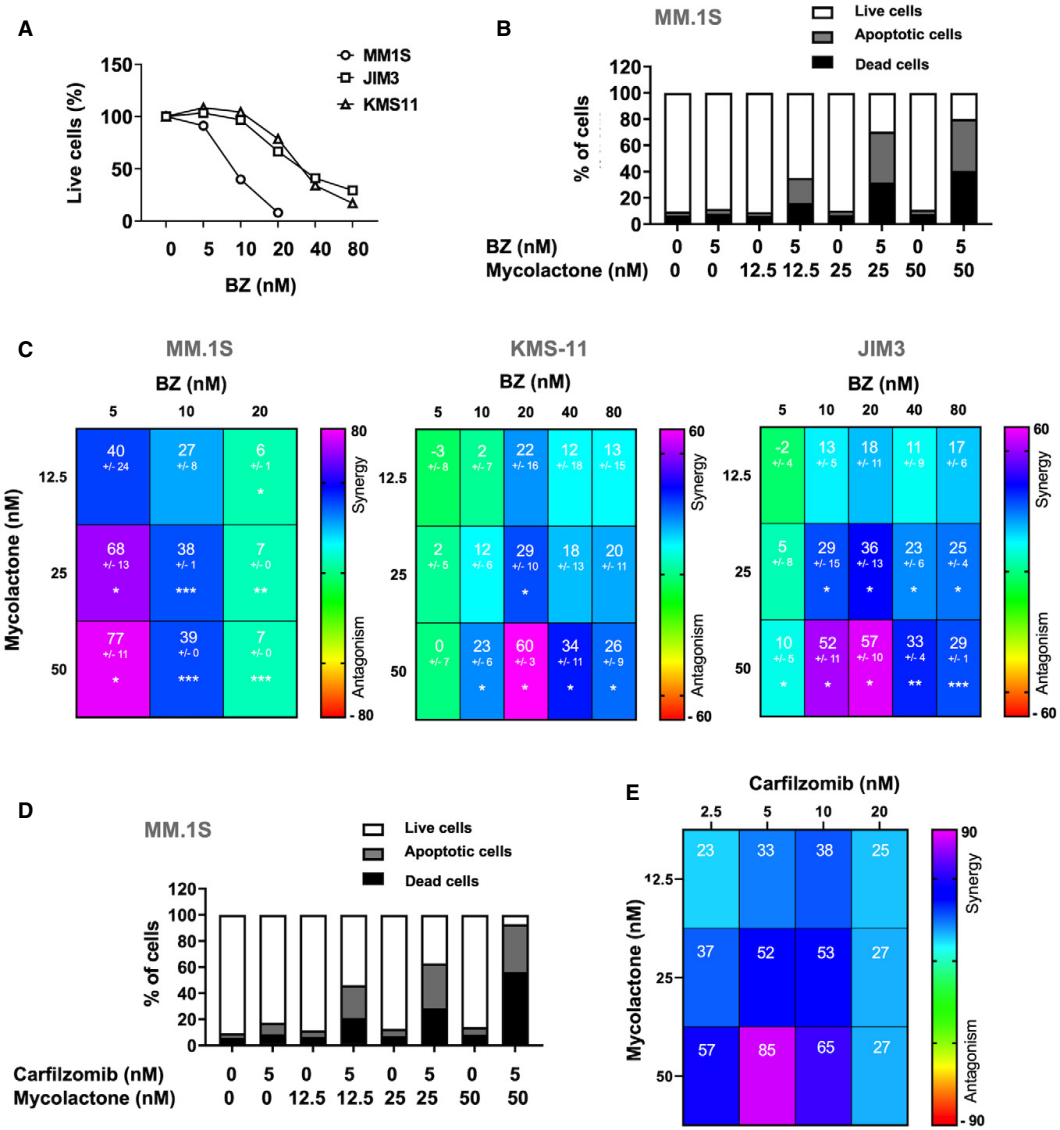
Mycolactone synergizes with PIs for induction of MM cell apoptosis AMM.1S, JIM3, and KMS‐11 cells were incubated with increasing concentrations of BZ for 24 h. The proportion of live (Annexin V^‐^ PI^‐^) cells was then measured by flow cytometry as in Appendix Fig S1. Data are Mean % of live cells from technical duplicates, relative to vehicle controls (Ctrl).BMM.1S were treated for 24 h with mycolactone and/or BZ at the indicated concentrations. Data are Mean % of live, apoptotic, and dead cells from technical duplicates, gated as in Appendix Fig S1, relative to total cells.CSynergy between mycolactone and BZ in the MM.1S, JIM3, and KMS‐11 cell lines, when treated as in (B). Data are Mean Loewe scores ± SD shown as heatmaps. *N* = 6 (cumulative data of three independent experiments with technical duplicates) for MM.1S and KMS‐11, and *N* = 4 (cumulative data of two independent experiments with technical duplicates) for JIM3. Statistical significance was assessed by Student’s *t*‐test: *P < 0.05; **P < 0.01; ***P < 0.001.D, EMM.1S cell were treated as in (B) but using carfilzomib instead of BZ (D). Synergy scores (E) were calculated as in (C). Data are Mean Loewe scores of technical duplicates, shown as heatmaps. Data information: In A and B, shown data are representative of two and three independent experiments with similar results, respectively.

**Figure EV1:**
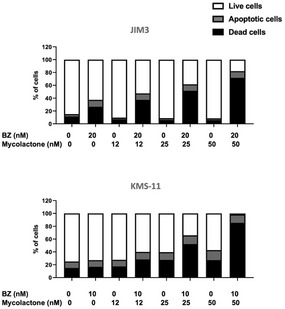
Mycolactone potentiates the effect of BZ in JIM3 and KMS‐11 JIM3 and KMS‐11 cells were treated with mycolactone and/or BZ at the indicated concentrations for 24 h. Data are Mean % of live, apoptotic, and dead cells from technical duplicates, gated as in Appendix Fig S1, relative to total cells. They are representative of two independent experiments with similar results.

The clinical efficacy of BZ is believed to result from its capacity to induce unresolvable proteotoxic stress in MM cells via accumulation of misfolded proteins in the cytoplasm (Gandolfi *et al*, 2017). Since Sec61 substrates blocked in translocation by mycolactone are diverted to the proteasome for degradation (Hall *et al*, 2014), we reasoned that mycolactone may potentiate the anti‐MM activity of BZ through generation of additional proteotoxic stress. While mycolactone‐mediated cytotoxicity did not manifest before 48 h of treatment (Fig 1A), BZ induced significant mortality in all MM cell lines after only 24 h (Fig 2A). Cell lines nevertheless differed in susceptibility to BZ cytotoxicity, JIM3 and KMS‐11 being relatively more resistant than MM.1S. To assess a potential synergy between mycolactone and BZ, MM cells were treated for 24 h with subtoxic doses of BZ together with increasing concentrations of mycolactone, and induction of apoptosis was assessed after 24 h. In all cell lines, the mycolactone–BZ combination induced more cell death than single drugs (Figs 2B and EV1). Heatmaps of synergy scores (Di Veroli *et al*, 2016) show that mycolactone synergized with BZ in all cell lines, irrespective of their basal resistance to BZ (Fig 2C). Similar findings were obtained with MM.1S cells treated with carfilzomib, a second‐generation PI that is proposed to BZ‐resistant MM patients (Fig 2D and E).

### The mycolactone–BZ combination activates pro‐apoptotic UPR in MM cells

**Figure 3:**
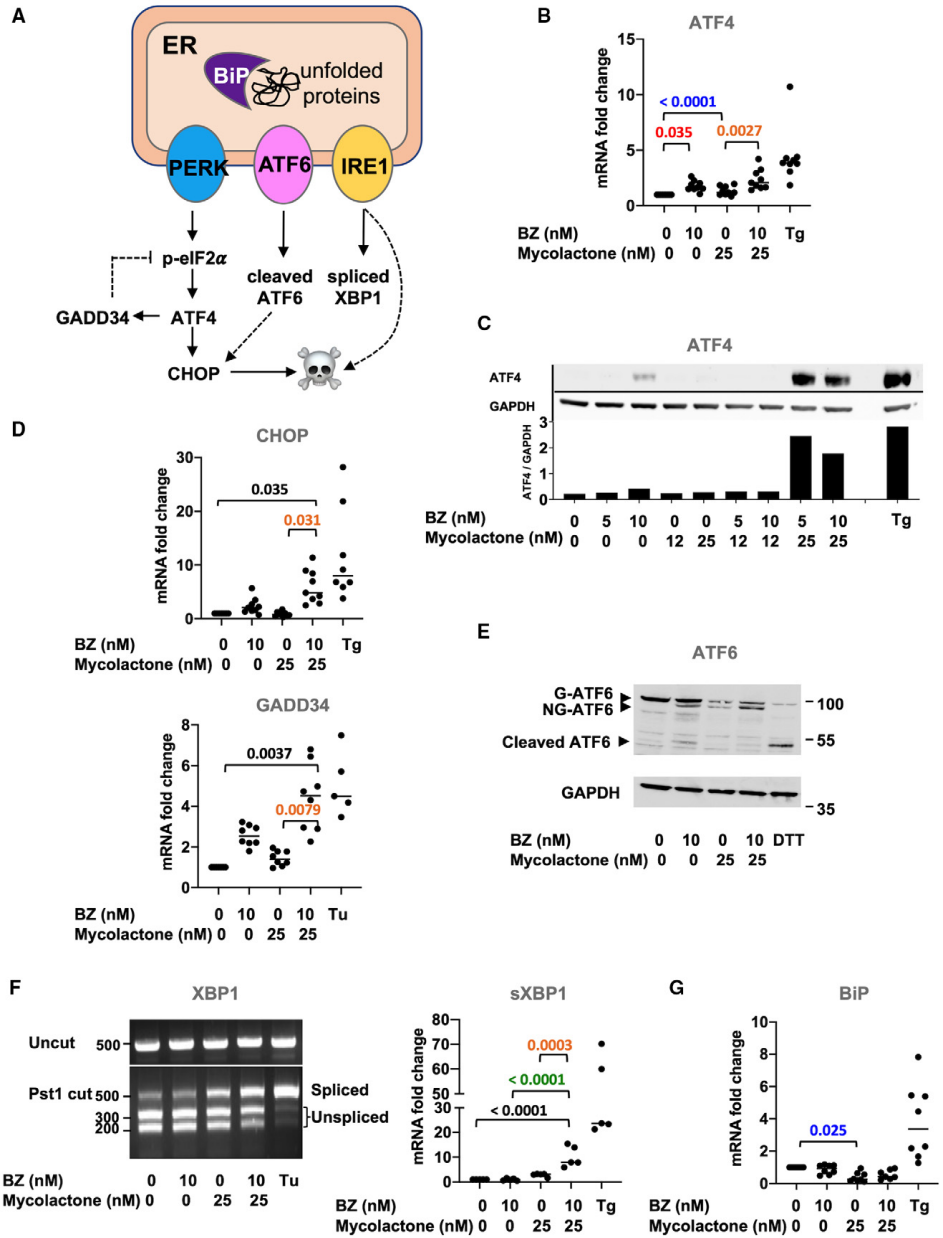
The mycolactone–BZ combination activates pro‐apoptotic UPR in MM.1S cells ADiagram illustrating the pro‐apoptotic pathways activated by UPR sensors PERK, ATF6, and IRE1 upon accumulation of unfolded proteins in the ER lumen.B–GIn MM.1S cells treated with mycolactone and/or BZ at the indicated concentrations, or vehicle as control for 6 h: (B) ATF4 mRNA levels were quantified by qPCR; (C) ATF4 protein levels in cell lysates were assessed by Western blot (top panel) and quantified relatively to GADPH levels (lower panel); (D) CHOP and GADD34 mRNA levels were quantified by qPCR; (E) ATF6 and GAPDH protein levels in cell lysates were assessed by Western blot with molecular weight markers (kDa) indicated on the right; Bands corresponding to glycosylated (G‐ATF6), non‐glycosylated (NG‐ATF6), and cleaved ATF6 are indicated by arrows; (F) Total RNA was isolated and used as a template for RT‐PCR of XBP‐1 (upper panel), which was then digested with Pst1 and separated on a 2% agarose gel (lower panel) with molecular weight markers (bp) indicated on the left; spliced XBP1 (sXBP1) mRNA levels were also quantified by qPCR and normalized to total (spliced + unspliced) XBP1 mRNA levels, and (G) BiP mRNA level was quantified by qPCR. Data information: Shown mRNA data are mean fold changes (2^−ΔΔCT^) ± SD, relative to untreated controls (cumulative data of at least two independent experiments with technical duplicates or triplicates), pairwise compared by nested one‐way ANOVA with Tukey’s multiple‐comparison test, exact *P*‐values indicated. Thapsigargin (Tg, 2 µM, 6 h), tunicamycin (Tu, 2 µM, 6 h), or DTT (4 mM, 2 h) were used as positive controls. In C, E, and F, shown data are representative of two independent experiments with similar results.

We next sought to determine if the synergistic induction of apoptosis in MM cell lines treated with mycolactone and BZ correlated with enhanced ER stress. The unfolded protein response (UPR) is activated by three ER‐resident transmembrane sensor proteins: protein kinase RNA‐like ER kinase (PERK), inositol requiring enzyme 1 (IRE1), and activating transcription factor (ATF) 6 (Fig 3A) (Hetz & Papa, 2018). Activation of PERK leads to the phosphorylation of eIF2α, with p‐eIF2α reprogramming protein translation to promote the expression of stress response mRNAs such as ATF4. ATF4 subsequently drives the expression of genes alleviating stress such as the growth arrest and DNA damage‐inducible protein (GADD34), which promotes p‐eIF2α dephosphorylation in a negative feedback loop. Activation of IRE1 triggers the splicing of X‐box‐binding protein 1 (XBP1) mRNA into its transcriptionally active form (sXBP1), able to transcriptionally reprogram UPR target genes. Chronic stress promotes transition from adaptive to terminal UPR, marked by ATF4‐mediated induction of pro‐apoptotic C/EBP homology protein (CHOP) expression and accentuated by pro‐death signals originating from IRE1 signaling. Activation of ATF6 leads to its cleavage into an active form in the Golgi, which contributes to upregulate CHOP.

**Figure EV2:**
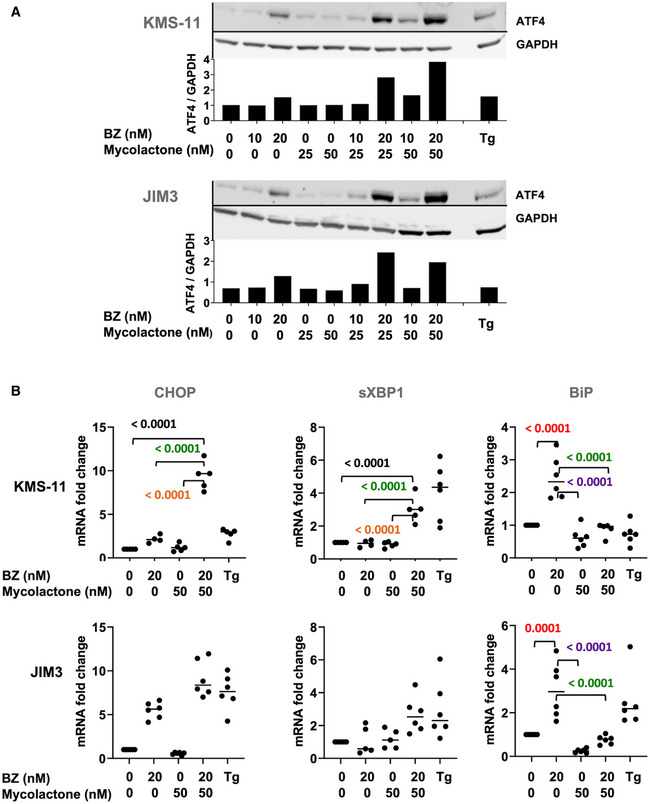
The mycolactone–BZ combination activates pro‐apoptotic UPR in KMS‐11 and JIM3 cells KMS‐11 and JIM3 cells were treated with mycolactone and/or BZ, or vehicle as control, for 6 h. ATF4 protein levels were assessed in cell lysates by Western blot (top panel) and quantified relative to GADPH levels (lower panel). Data are representative of two independent experiments with similar results.CHOP, sXBP1, and BiP mRNA levels were quantified by qPCR in the two cell lines treated as in (A). sXBP1 mRNA levels were normalized to total (spliced + unspliced) XBP1 mRNA level. Data are Mean RNA fold changes (2^−ΔΔCT^) ± SD, relative to untreated controls. *N* ≥ 5 (cumulative data of two independent experiments with technical duplicates and triplicates), pairwise compared by nested one‐Way ANOVA with Tukey’s multiple‐comparison test, exact *P*‐values indicated. Thapsigargin (Tg, 2 µM, 6 h) was used as a positive control. Source data are available online for this figure.

In all MM cell lines treated with subcytotoxic concentrations of mycolactone and BZ for 6 h, we detected an increased expression of ATF4, at both the mRNA and protein levels, compared to single drug treatments (Figs 3B and C, and EV2A). Elevated ATF4 correlated with enhanced expression of its CHOP target (Figs 3D and EV2B), reflecting the activation of pro‐apoptotic eIF2α/ATF4/CHOP signaling. It was also associated with the induction of ATF4 target GADD34 (Fig 3D), suggesting that dephosphorylation of p‐eIF2α is induced in treated MM cells. In MM.1S cells treated with BZ for 6 h, we also observed defects in ATF6 glycosylation and partial cleavage of the protein (Fig 3E). In agreement with previous reports (Ogbechi *et al*, 2018), mycolactone did not induce such ATF6 cleavage. However, defective glycosylation and depletion of full‐length ATF6 were observed, likely resulting from mycolactone‐mediated blockade of this Type II transmembrane protein in translocation. MM1.S cells exposed to the mycolactone–BZ combination displayed the sum of the alterations induced by each drug, namely depletion, defective glycosylation, and partial cleavage of ATF6. Finally, gel electrophoresis and qPCR analyses demonstrated an enhanced XBP1 splicing in MM1.S cells treated with the mycolactone/BZ combination, compared to single drugs (Fig 3F). A significant upregulation of sXBP1 mRNA levels was also observed in KMS‐11 cells exposed to the drug combination (Fig EV2B). From these data, we conclude that the mycolactone–BZ combination hyperactivates PERK and IRE1 signaling in MM cells, with potent activation of the ATF4/CHOP axis indicating a transition to terminal UPR. The ER‐resident chaperone BiP is a master regulator of the UPR that is normally induced by ER stress and determines the threshold for induction of apoptosis (Hetz & Papa, 2018). It is interesting to note that BiP expression was not augmented in MM cells treated with mycolactone, alone or combined to BZ (Figs 3G and EV2B).

### The cytotoxic synergy between mycolactone and BZ is maintained in BZ‐resistant MM.1S cells

**Figure 4:**
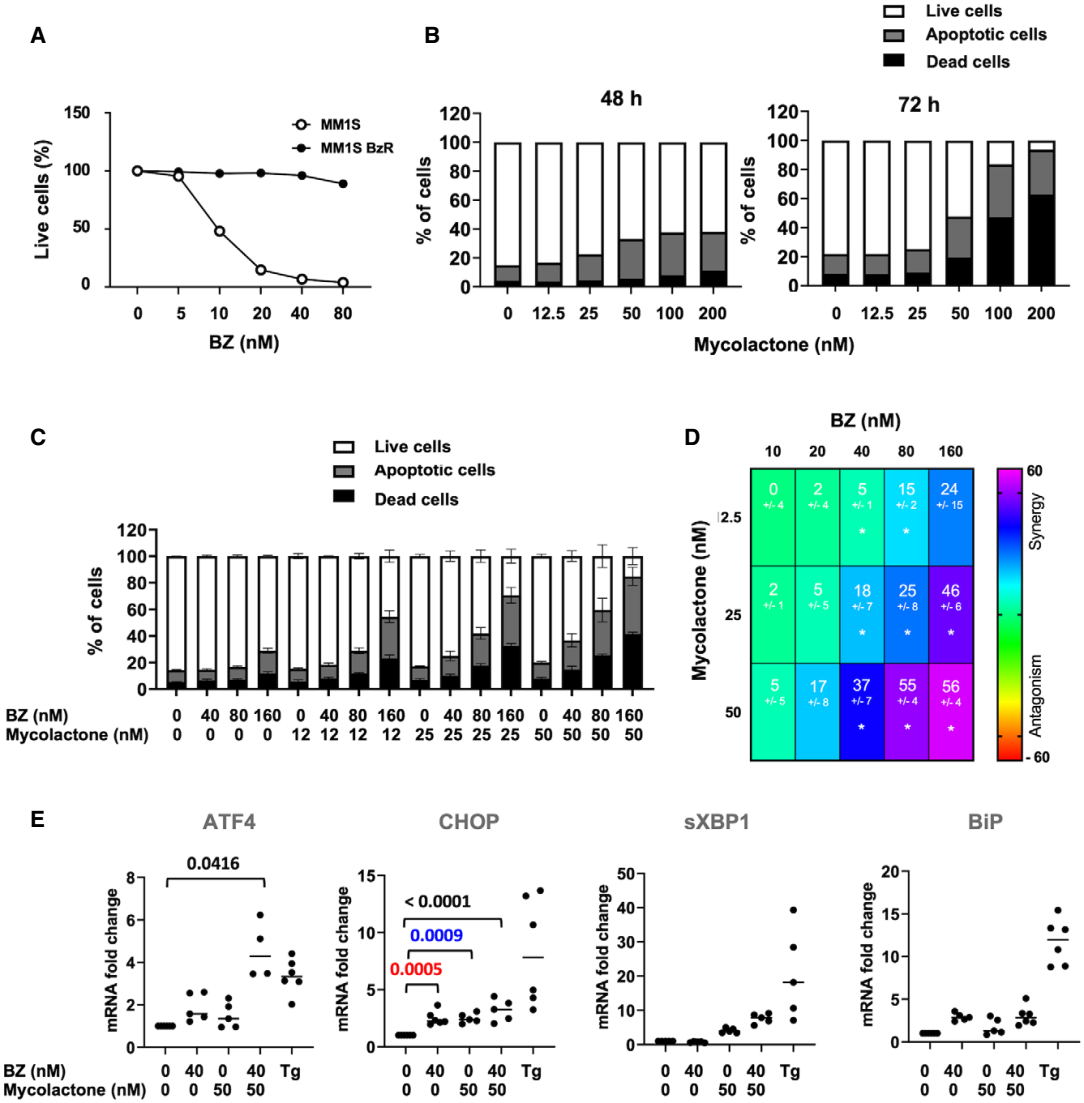
The cytotoxic synergy between mycolactone and BZ is maintained in BZ‐resistant cells Parental MM.1S (BzS) and MM.1S BzR cells were incubated with increasing concentrations of BZ for 24 h. The proportion of live (Annexin V^‐^ PI^‐^) cells was then measured by flow cytometry as in Appendix Fig S1. Data are Mean % of live cells from technical duplicates, relative to vehicle controls.Effect of mycolactone on MM.1S BzR cell viability after 48 and 72 h of treatment, as measured by exposure of Annexin V and incorporation of PI. Data are Mean % of live, apoptotic, and dead cells from technical duplicates, gated as in Appendix Fig S1, relative to total cells.MM.1S BzR cells were treated for 24 h with mycolactone and/or BZ at the indicated concentrations, or vehicle as control. Data are Mean % ± SD of live, apoptotic, and dead cells, gated as in Appendix Fig S1, relative to total cells. *N* = 6 (cumulative data of three independent experiments with technical duplicates).Synergy between mycolactone and BZ in the MM.1S BzR lines, when treated as in (C). Data are Mean Loewe scores ± SD, shown as heatmaps. Differences between treated cells and controls by Student’s *t*‐test: *P < 0.05; *N* = 6 (cumulative data of three independent experiments with technical duplicates).ATF4, CHOP, sXBP1, and BiP mRNA levels were quantified by qPCR in MM.1s BzR cells treated as in (C). sXBP1 mRNA levels were normalized to total (spliced + unspliced) XBP1 mRNA level. Data are Mean RNA fold changes (2^−ΔΔCT^) ± SD, relative to untreated controls. *N* = 5 (cumulative data of two independent experiments with technical duplicates and triplicates), pairwise compared by nested one‐way ANOVA with Tukey’s multiple‐comparison test, exact *P*‐values indicated. Thapsigargin (Tg, 2 µM, 6 h) was used as a positive control. Data information: In A and B, shown data are representative of two independent experiments with similar results.

The development of resistance to PIs is a major obstacle to successful treatment of MM patients (Lawasut *et al*, 2012). To determine if the cytotoxic synergy between mycolactone and BZ was maintained in BZ‐resistant MM cells, we generated a BZ‐resistant version of the MM.1S cell line (MM.1S BzR cells), with stable and > 15× increased resistance to BZ treatment (Fig 4A). Notably, MM.1S BzR cell susceptibility to mycolactone was identical to that of MM.1S BzS (Figs 4B and 1A), showing that acquired resistance to BZ does not confer cross‐resistance to mycolactone. As in MM.1S BzS cells (Fig 2C), mycolactone synergized with BZ for induction of MM.1S BzR cell death (Fig 4C and D) and engagement of apoptosis in MM.1S BzR cells exposed to the mycolactone–BZ combination correlated with activation of ER stress responses, reflected by elevated levels of ATF4, CHOP, and sXBP1 mRNAs (Fig 4E). Like MM.1S BzS cells, MM.1S BzR cells failed to upregulate BiP expression upon exposure to the mycolactone/BZ drug combination (Fig 4E). Together, these data demonstrate that mycolactone cytotoxicity and synergy with BZ override MM cell resistance to BZ. They consolidate the clinical relevance of Sec61 inhibitor‐based treatments for MM.

### The synergy between mycolactone and BZ cytotoxicity extends to mouse B‐cell lymphomas

**Figure 5:**
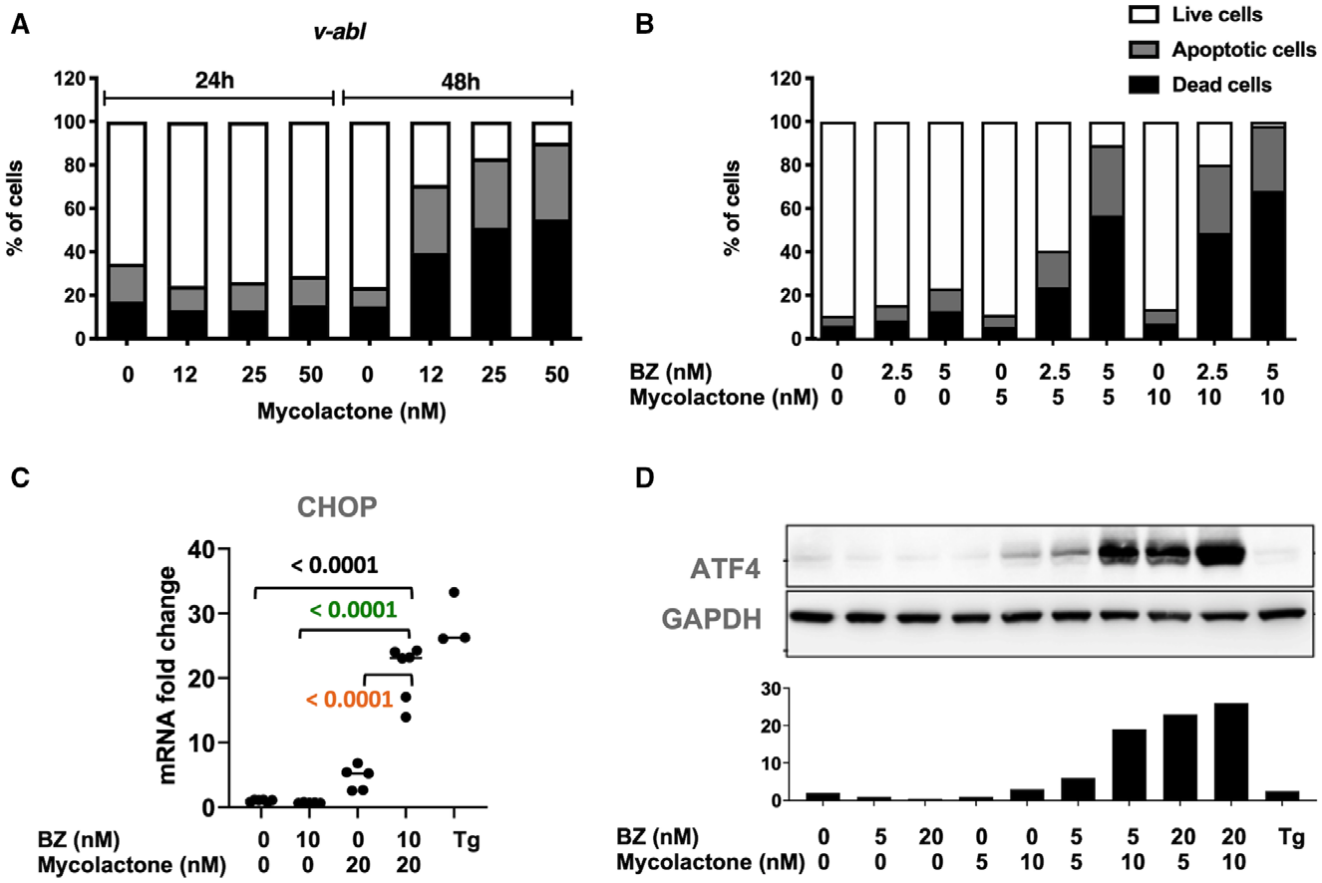
The synergy between mycolactone and BZ cytotoxicity extends to mouse B‐cell lymphomas Effect of mycolactone on v‐abl pro‐B‐cell viability after 24 and 48 h of mycolactone treatment, as measured by exposure of Annexin V and incorporation of PI. Data are Mean % of live, apoptotic, and dead cells from technical duplicates, gated as in Appendix Fig S1, relative to total cells.v‐abl pro‐B cells were treated for 24 h with mycolactone and/or BZ at the indicated concentrations. The percentage of live, apoptotic, and dead cells was then measured by flow cytometry as in Appendix Fig S1. Data are Mean % from technical duplicates, relative to total cells.v‐abl pro‐B cells were treated with mycolactone and/or BZ at the indicated concentrations for 6 h. CHOP mRNA levels were quantified by qPCR. Data are Mean RNA fold changes (2^−ΔΔCT^) ± SD relative to untreated controls. *N* = 6 (cumulative data of three independent experiments with technical duplicates), pairwise compared by nested one‐way ANOVA with Tukey’s multiple‐comparison test, exact *P*‐values indicated.v‐abl pro‐B cells were treated for 6 h with mycolactone and/or BZ at the indicated concentrations. ATF4 protein levels were assessed in cell lysates by Western blot (top panel) and quantified relative to GADPH levels (lower panel). Data information: Data shown in A, B, and D are representative of experiments performed with two independent v‐abl cell clones, with similar results. In C and D, thapsigargin (Tg, 2 µM, 6 h) was used as a positive control.

Besides MM, PIs show promise in the treatment of other hematological malignancies such as acute leukemia (Cloos *et al*, 2017) and solid malignancies (Roeten *et al*, 2018). We tested the proteotoxic effects of mycolactone, alone and combined with BZ, in B‐cell acute lymphoblastic leukemia (B‐ALL) using mouse pro‐B‐cell lines generated by transformation of hematopoietic cells with the murine viral form of the Abelson oncogene (v‐abl). Mycolactone alone demonstrated potent ability to induce v‐abl cell apoptosis *in vitro* (Fig 5A). Moreover, as in human MM cell lines, it synergized with BZ for induction of v‐abl cell death (Fig 5B) and this correlated with activation of the ER stress‐associated ATF4/CHOP apoptotic pathway (Fig 5C and D). In addition to illustrating mycolactone’s ability to efficiently block mouse Sec61, these data revealed the potential interest of Sec61 inhibitors for the treatment of B‐ALL, and potentially other proteasome inhibition‐susceptible malignancies.

### Patient‐derived MM tumors are highly susceptible to mycolactone toxicity and synergy with BZ

**Figure 6:**
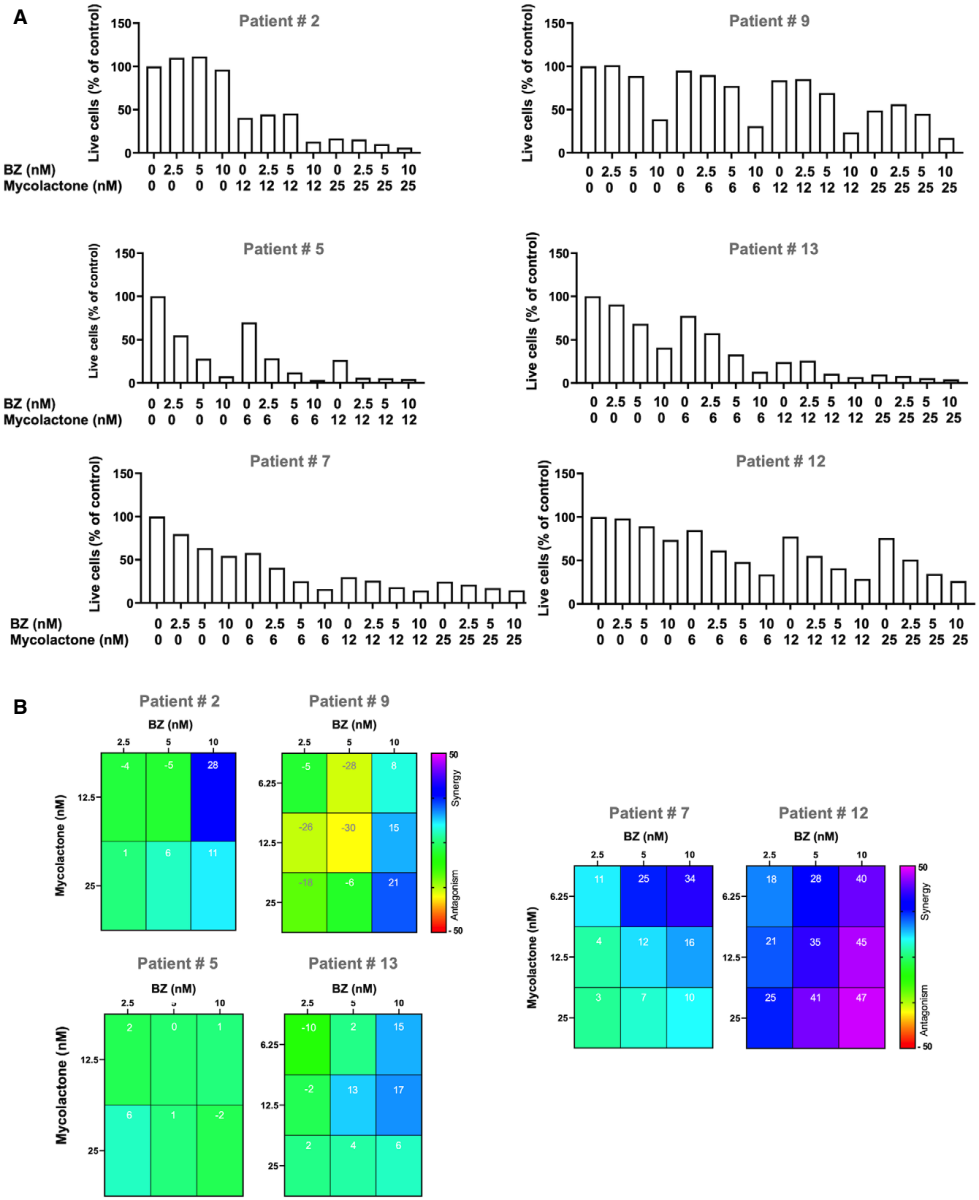
MM primary tumors are highly susceptible to mycolactone toxicity and synergy with BZ Mononuclear cells from bone marrow aspirates of newly diagnosed (#2, #5, #9, and #13) or relapsed (#7 and #12) MM patients were treated with mycolactone and/or BZ at the indicated concentrations, or vehicle as control, for 18 h. Then, MM cells were identified by staining with anti‐CD38 and anti‐CD138 antibodies using the gating strategy depicted in Appendix Fig S2.
Following treatment, induction of apoptosis/necrosis was measured by exposure of annexin V and PI incorporation. Data are Mean % of live cells from technical duplicates, relative to controls.Synergy between mycolactone and BZ in treated tumors. Data are Mean Loewe scores from technical duplicates, shown as heatmaps.

**Figure EV3:**
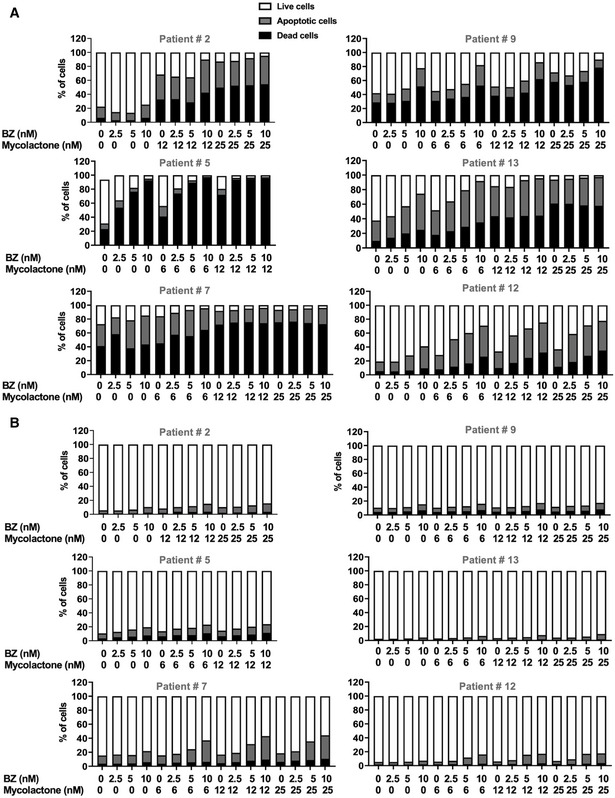
Differential toxicity of mycolactone–BZ combination in MM cells and non‐cancerous lymphoid cells in bone marrow aspirates A, BIncidences of live, apoptotic, and dead cells within the MM cell (A) and the non‐cancerous lymphoid cell (B) subsets are compared in newly diagnosed (#2, #5, #9, and #13) or relapsed (#7 and #12) MM patients after an 18 h treatment with mycolactone and/or BZ. MM cells (A) and non‐cancerous lymphoid cells (B) were identified with the gating strategy depicted in Appendix Fig S2. Data are Mean % of live, apoptotic, and dead cells from technical duplicates, relative to total cells.

We next assessed the anti‐MM activity of mycolactone, alone and combined to BZ, in patient‐derived tumors. Mononuclear cells were isolated from bone marrow aspirates of four newly diagnosed MM patients, and two patients with relapsed MM after at least one line of treatment including PI and IMiDs (Table 1). Cells were placed in culture medium within 3 h post‐biopsy, then exposed to mycolactone and/or BZ for 18 h. Induction of apoptosis was determined in MM cells, gated as CD38^+^ CD138^+/−^ plasma cells, following Annexin V/PI staining (Appendix Fig S2). Figure 6A shows the results as % of live MM cells and Fig EV3A as % of live/apoptotic/dead cells, for easier comparison with treatment effect on non‐cancerous lymphoid cells (Fig EV3B). MM cells from the six studied patients varied in susceptibility to BZ treatment, irrespective of their newly diagnosed or relapsed status (Figs 6A and EV3A). Tumor sensitivity to mycolactone was also variable, and not systematically associated with sensitivity to BZ (Figs 6A and EV3A). In all studied patients, significant cell death was achieved with 18 h exposure to ≥ 12 nM mycolactone, an anti‐MM activity globally equivalent to that of 10 nM BZ. Strikingly, mycolactone synergized with BZ to kill MM cells from all patients irrespective of their treatment naive or relapsed status and relative resistance to single drugs (Fig 6B), further demonstrating that mycolactone‐mediated cytotoxicity operates irrespective of MM cell resistance to BZ.

**Table 1:** Characteristics of the MM patients included in this study.

Patient	P2	P5	P7	P9	P12	P13
Age (years)	57	72	72	67	72	66
Gender (F/M)	F	F	F	F	M	F
Years since diagnosis (years)	–	–	10	–	5	–
Lines of treatment	–	–	2	–	1	–
Treatments						
IMiDs	–	–	2	–	1	–
PI	–	–	2	–	1	–
Alkylating agent	–	–	1	–	1	–
HSCT^a^	–	–	1	–	–	–

^a^ Hematopoietic stem cell transplantation.

**Figure EV4:**
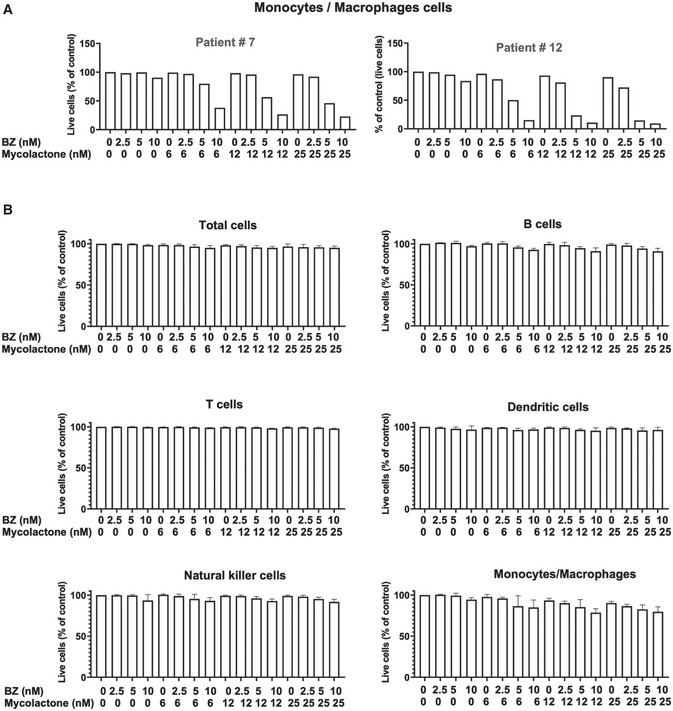
Toxicity of the mycolactone–BZ combination in PBMCs and bone marrow monocytes/macrophages The proportion of live (Annexin V^‐^ PI^‐^) cells within the monocyte/macrophage subset of mononuclear cells, after a 18 h treatment with mycolactone and/or BZ is shown for the two relapsed patients. Mononuclear cells from bone marrow aspirates were treated as in Fig 6. The monocyte/macrophage subset was identified using the gating strategy depicted in Appendix Fig S2. Data are Mean % of live cells, relative to vehicle controls.PBMCs from healthy donors were treated with mycolactone and BZ, alone or in combinations, at the indicated concentration for 18 h. Cells were then labelled with fluorophore‐conjugated anti‐CD3, anti‐CD19, and anti‐CD11c. Total cells, B cells, T cells, dendritic cells, NK cells, and monocytes/macrophages were identified by flow cytometry analysis, using the strategy depicted in Appendix Fig S3. Data are Mean % ± SD of live cells, relative to vehicle controls. *N* = 6, cumulative data from three donors with technical duplicates.

Importantly, the cytotoxicity of the mycolactone–BZ combination selectively affected MM cells, as minimal cell death was recorded in CD38^‐^ CD138^‐^ cells from the same bone marrow aspirates (Fig EV3A and B). It is noteworthy that bone marrow aspirates from the two relapsed patients contained a relatively higher incidence of monocytes/macrophages, as characterized by their SSC/FSC profile (as shown for Patient #7 in Appendix Fig S2). Although drugs alone had little effect, the mycolactone–BZ combination displayed some toxicity on this cell subset at the highest tested concentrations (Fig EV4A). To further assess the impact of mycolactone–BZ combination on immune cell viability, we subjected peripheral blood mononuclear cells (PBMCs) to the same drug treatments as patient‐derived tumors (Fig 6 Appendix Fig S3). Minimal or no viability loss was observed in total PBMCs, nor in gated T cells, B cells, natural killer cells, dendritic cells, and monocytes/macrophages exposed to mycolactone, BZ, or both drugs (Fig EV4B), indicating that the cytotoxicity of the mycolactone–BZ combination is selective of MM cells.

### Combining mycolactone with BZ delays MM tumor growth *in vivo*

**Figure 7:**
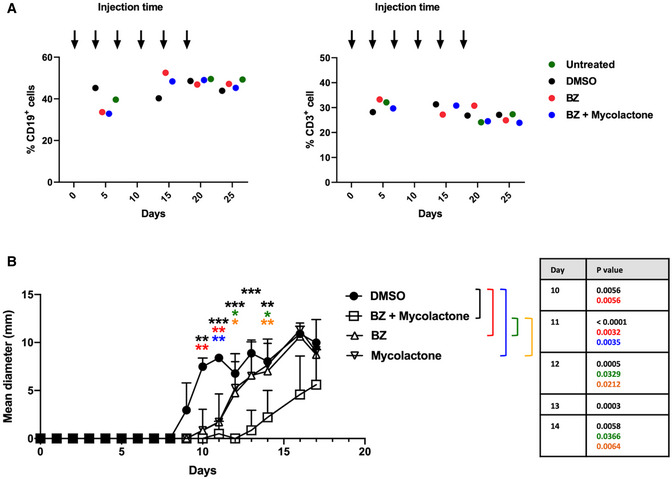
Combining mycolactone with BZ delays MM tumor growth *in vivo* C57BL/6 mice (*N* = 2/group) were injected intraperitoneally with BZ (0.5 mg/kg), BZ (0.5 mg/kg) + mycolactone (0.3 mg/kg), DMSO as vehicle controls every 3.5 days, or left untreated. Blood samples were analyzed 5, 15, 20, and 25 days after the first injection and percentages of CD3^+^ and CD19^+^ cells determined by flow cytometry. Results are expressed as % of positive cells among total mononuclear cells, each dot representing the Mean % from two mice.NSG mice (*N* = 9/group) were injected subcutaneously with 3 × 10^6^ MM.1S cells at day 0. Seven days later, mice were injected with DMSO, BZ (0.5 mg/kg), and/or mycolactone (0.3 mg/kg) every 3.5 days intraperitoneally and tumor growth was followed by daily measurement of the tumor diameter. Data are Mean tumor diameters ± SD and represent cumulative data from three independent experiments with 3 mice/group. Difference between groups were analyzed with Tukey’s multiple‐comparison test using two‐way ANOVA, with mixed‐effect model for each time points: *P < 0.05; **P < 0.01; ***P < 0.001 (exact *P*‐values indicated). Only significant differences are shown.

To evaluate the therapeutic efficacy of the mycolactone–BZ combination *in vivo*, we first analyzed its toxicity in mice. C57BL/6 mice were treated twice a week by intraperitoneal injection of BZ (0.5 mg/kg) alone and/or mycolactone (0.3 mg/kg) for 3 weeks, BZ and mycolactone treatments previously shown to induce anti‐MM activity (LeBlanc *et al*, 2002) and anti‐inflammatory effects in injected mice (Guenin‐Mace *et al*, 2015), respectively. No sign of distress or weight loss could be detected in mice receiving single drugs or the drug combination, and their blood cell counts remained unaltered throughout the experiment (Fig 7A), illustrating the safety of these treatment regimen. We next compared the anti‐MM activity of mono‐ and bi‐therapies in immunodeficient NOD/SCID/IL2rγ^null^ (NSG) mice. Mice were subcutaneously engrafted with MM.1S cells, and 7 days later they were assigned to four treatment groups receiving (i) 0.5 mg/kg BZ, (ii) 0.3 mg/kg mycolactone, (iii) both drugs at these concentrations, or (iv) vehicle, twice weekly via the intraperitoneal route. Under these conditions, BZ and mycolactone both induced a minor yet significant delay in tumor growth, compared to vehicle controls (Fig 7B). Notably, the mycolactone–BZ combination was superior to single drug treatments in slowing down MM development. In addition to revealing a therapeutic window for Sec61 blockade in MM, these data confirmed the interest of combining inhibitors of translocon and proteasome in MM therapy.

## Discussion

Although previous studies have suggested the interest of targeting Sec61 in Oncology, therapeutic indications and *in vivo* safety of Sec61 blockers as anti‐cancer agents have remained unexplored (Luesch & Paavilainen, 2020). The first identified Sec61 inhibitor, cotransin, was isolated by screens for molecules decreasing the expression of cell adhesion molecules by a vascular endothelial cell line (Garrison *et al*, 2005). The high substrate selectivity of cotransin was subsequently used to specifically block cancer cell expression of HER3 (Ruiz‐Saenz *et al*, 2015), a receptor activating oncogenic signaling. Here, we took the opposite approach and sought to exploit the broad, almost total lack of selectivity of mycolactone for Sec61 substrates (Morel *et al*, 2018). Having shown that Sec61 blockade by mycolactone elicits potent ER stress responses (Morel *et al*, 2018), we selected the MM cancer model for proof‐of‐concept because of its particularly high susceptibility to proteotoxic stress (Vincenz *et al*, 2013). Furthermore, we knew from previous studies that systemically delivered mycolactone diffuses broadly in injected organisms to accumulate in leukocytes of peripheral blood and lymphoid organs (Hong *et al*, 2008; Phillips *et al*, 2009), a distribution profile that was adapted to plasma cell targeting. Finally, we reasoned that mycolactone may cause defects in MM cell expression of membrane receptors critically contributing to its dissemination and growth, potentially providing an additional therapeutic benefit *in vivo*. Our *in vitro* investigations using MM cell lines validated these predictions. Moreover, our observations that mycolactone efficiently kills tumors from treatment naive and relapsed MM patients *ex vivo* and delays MM xenograft growth in a pre‐clinical murine model of disease establish Sec61 as a novel therapeutic vulnerability in MM.

Serum levels of paraproteins, the antibody fragments produced by MM, are used clinically to diagnose MM and monitor its progression (Tate, 2019). In MM cell lines, the level of immunoglobulin light chains released in culture supernatant was dose dependently decreased by mycolactone after 24 h, and this inhibition closely correlated with the onset of apoptosis at 72 h. This result is in line with our previous demonstration that inhibition of protein secretion on the one hand and induction of cell apoptosis on the other hand are early and late effects of mycolactone‐mediated Sec61 blockade, respectively (Guenin‐Macé *et al*, 2019; Demangel, 2021). This suggests that the prognostic significance of serum paraproteins would be conserved in MM patients treated with Sec61 blockers. Further, mycolactone‐mediated inhibition of paraprotein secretion may help limit their pathophysiological effects in treated patients (Cook & Macdonald, 2007).

The introduction of PIs in anti‐MM chemotherapies has significantly improved patient survival and PIs now form one of the backbones of treatment (Kouroukis *et al*, 2014). It was therefore important to determine whether Sec61 blockade interferes with the anti‐tumor activity of PIs. Our data obtained in MM cell lines revealed a synergistic effect between mycolactone and BZ for induction of terminal UPR, with combination of subtoxic concentrations of each drug triggering MM cell death within 24 h. Similar results were obtained with Carfilzomib, indicating that our findings obtained with BZ generalize to second‐generation PIs. The mycolactone–BZ combination was superior to single drugs in killing patient‐derived MM tumor cells *ex vivo* and delaying MM growth in mouse models of disease, demonstrating the interest of targeting both the translocon and the proteasome in MM. Most importantly, mycolactone cytotoxicity and synergy with BZ were observed in isogenic BZ‐susceptible and ‐resistant MM cells. This indicates that mycolactone and BZ resistance mechanisms do not overlap and suggests that MM patients developing resistance to PIs will respond to Sec61 blockade therapy.

Transition from adaptative to terminal UPR is primarily triggered by induction of ATF4/CHOP signaling (Hetz & Papa, 2018), which was potently activated by the BZ–mycolactone combination in all studied MM cell lines. Surprisingly, while expression of p‐eIF2α target ATF4 was clearly induced in MM.1S cells treated with the BZ–mycolactone combination, we could not demonstrate enhanced phosphorylation of eIF2α by Western blot analysis of cell lysates. Our observation that GADD34 expression was activated at the same time as CHOP indicates that drug treatment keeps p‐eIF2α dephosphorylation active but fails to inhibit the eIF2α/ATF4/CHOP pathway. In MM cells, high secretory activity associated with intracellular accumulation of misfolded immunoglobulin chains is believed to account for the limited ability of BiP to cope with PI‐mediated ER stress, and the particularly low threshold for induction of terminal UPR upon exposure to PIs in these cells (Obeng *et al*, 2006). In support of this hypothesis, recent genome‐wide meta‐analysis of differentially expressed genes indicated that *Sec61A1* transcript levels are significantly upregulated in MM versus normal plasma cells (Katiyar *et al*, 2021). We reported previously that mycolactone‐mediated UPR differs from that triggered by typical ER stressors by the lack of BiP induction (Baron *et al*, 2016). In line with this finding, despite displaying clear marks of ER stress, MM cell lines failed to upregulate BiP expression upon exposure to mycolactone (Figs 3, 4 and EV2). In all cell lines, this defect was maintained in the presence of BZ. Therefore, we propose that mycolactone sensitizes MM cells to BZ‐driven apoptosis by preventing BiP induction.

Today, the gold standard of MM care is a combination of PIs, IMiDs, and corticosteroids. While the majority of newly diagnosed patients respond to this three‐drug combination, all eventually develop drug resistance. IMiDs such as lenalidomide operate primarily by targeting Cereblon (CRBN) (Zhu *et al*, 2019), a substrate receptor of the CRL4 ubiquitin ligase complex. IMiDs binding to CRBN leads to increased proteasomal degradation of pro‐survival transcription factors IKZF1/3, thereby promoting MM apoptosis. Notably, low CRBN expression is often associated with IMiD resistance and functional introduction of CRBN mutations in MM cells conferred resistance to lenalidomide *in vitro* (Kortüm *et al*, 2016). In both MM cell lines and primary tumors, mycolactone displayed cytotoxic effects irrespective of the cell basal resistance to BZ. It will be interesting to determine whether mycolactone also kills IMiD‐resistant MM and synergizes with IMiDs for induction of MM cell death.

Similar to PIs and IMiDs, the clinical use of Sec61 inhibitors raises questions about their potential toxicity in normal cells, and the risks to generate drug resistance in tumor cells. Mycolactone‐mediated Sec61 blockade provokes perturbations in protein homeostasis whose magnitude and ability to evolve toward apoptosis vary across cell types, depending on their functional biology (Demangel & High, 2018; Morel *et al*, 2018). Recent studies using computer simulations or lipid monolayers also suggested that mycolactone passage across cellular membranes may alter their integrity and dynamic properties (López *et al*, 2018; Nitenberg *et al*, 2018). Using primary human cells, we reported previously that contrary to macrophages, neutrophils and T lymphocytes are resistant to mycolactone‐mediated toxicity (Guenin‐Mace *et al*, 2015). The present study further indicates that MM cells are more susceptible to Sec61 blockade‐driven apoptosis than major PBMC subsets. In mice, repeated systemic administration of mycolactone significantly delayed MM growth without inducing toxic effects. Together, our *in vitro* and *in vivo* results thus define a therapeutic window for Sec61 blockers in MM treatment. Regarding the development of resistance in tumor cells, one can speculate that increased expression of Sec61α may reduce the inhibitor’s ability to saturate Sec61‐binding sites, thereby the induction of ER stress responses. Sec61α is a mycolactone‐sensitive Sec61 client that was mildly upregulated by mycolactone in previous proteomic studies using various cancer cell lines (Grotzke *et al*, 2017; Morel *et al*, 2018), suggesting that UPR‐mediated increase in Sec61α gene expression may override Sec61 blockade, at least to a limited extent. Mutations in the Sec61α gene sequence resulting in loss of inhibitor binding, such as those introduced experimentally in cancer cell lines in previous research (Baron *et al*, 2016; Gérard *et al*, 2020), may also confer MM tumors with resistance to Sec61 inhibitors. To our knowledge, none of these mutations has been reported in human clinical samples, suggesting that they may prevent the translocon from ensuring essential functions. Yet, it will be important to verify if such genetic adaptations arise in MM cells subjected to Sec61 blocker pressure *in vitro* and in animal models of disease. Cytogenetic abnormalities are found in most MM patients, among which translocation t(4;14) that has been associated with poor prognosis (Abdallah *et al*, 2020). It is interesting to note that mycolactone was relatively more toxic in t(4;14)‐positive JIM3 and KMS‐11 cells than in t(4;14)‐negative MM.1S cells (Fig 1A). While a direct comparison between isogenic t(4;14)‐positive and ‐negative cell lines would be required to conclude, this result suggests that gene deregulation caused by NSD2 overexpression and expansion of H3K36me2 in t(4;14) MM cells (Lhoumaud *et al*, 2019) may confer them with enhanced susceptibility to the pro‐death programs triggered by Sec61 blockade.

To address the toxicity and resistance issues associated with PI‐based treatments, novel approaches using antibody–drug conjugates, bispecific T‐cell engager antibodies, and chimeric antigen receptor (CAR)‐T cells are in test phase. Such strategies target membrane receptors that are specifically expressed by MM cells, such as BCMA (Shah *et al*, 2020). Inhibiting Sec61 with cotransin increased the surface expression of BCMA by MM cell lines, resulting in enhanced efficacy a BCMA‐targeting antibody–drug conjugate (Ramkumar *et al*, 2020). In line with this finding, mycolactone dose dependently increased BCMA expression in the MM cell lines tested in the present study (Fig 1). This argues that anti‐MM therapies targeting BCMA may benefit from combination with mycolactone.

Here, we used immunodeficient NSG mice to assess the translational potential of mycolactone. Although this setting is widely used to estimate the therapeutic index of drug candidates, it does not allow to evaluate their impact on immune responses. Since Sec61 inhibition strongly affects the functional biology of immune cells (Guenin‐Mace *et al*, 2015; Baron *et al*, 2016; Grotzke *et al*, 2017; Demangel & High, 2018; Guenin‐Macé *et al*, 2019; Demangel, 2021), it may suppress the development of anti‐tumor immunity. Further investigations using immunocompetent mouse models of MM and B‐ALL will be needed to address this hypothesis. Regardless of this limitation, this study reports the first attempt to combine inhibitors of both proteasome and translocon in Oncology. Our results establish Sec61 blockade as a novel therapeutic approach in MM, synergizing with proteasome inhibition. We used mycolactone for proof of concept, as it displays unprecedented Sec61 inhibition potency and a body‐wide distribution *in vivo* (Guenin‐Macé *et al*, 2019). However, mycolactone is a complex natural product whose chemical synthesis is a costly, multi‐step process making large‐scale industrial production challenging (Song *et al*, 2007; Gehringer & Altmann, 2017; Saint‐Auret *et al*, 2017). Structure–activity relationship studies have identified the minimal structural module retaining Sec61‐binding activity in mycolactone (Guenin‐Mace *et al*, 2015; Baron *et al*, 2016) and the tri‐dimensional structure of the Sec61 complex inhibited by mycolactone was recently resolved (Gérard *et al*, 2020). Our study thus paves the way to structure‐based design of drug‐like surrogates of mycolactone as novel therapeutics for MM, and potentially other proteostasis‐addicted cancers, such as non–small cell lung cancers and certain solid tumors.

## Materials and Methods

### Patients

Patients were seen at Saint‐Louis hospital, Paris, France, for a new or known diagnosis of MM. Characteristics of the patients are shown in Table 1. Bone marrow aspirates were performed in the Department of Immuno‐Hematology of the Saint‐Louis Hospital using standard procedures, as part of routine diagnostic work‐up. Written informed consent was obtained from all patients, and experiments conformed to the principles set out in the WMA Declaration of Helsinki and the Department of Health and Human Services Belmont Report. All samples containing measurable numbers of viable mononuclear cells were processed and included in data analysis. The protocol received approval of the ethical committee “Groupements hospitaliers universitaires” GHU Nord; No. 23798‐2020012709469025 # 121.

### Reagents

Mycolactone was purified from *M. ulcerans* bacterial pellets (strain 1615, ATCC 35840), then quantified by spectrophotometry, and stored in ethanol at −20°C protected from light. For *in vivo* experiments, a 4 mM stock was diluted in a NaCl solution (0.9% w/v) immediately before injection in animals. For *in vitro* experiments, a 1,000× working solution was prepared by dilution of the ethanol stock in DMSO and stored at −20°C, then thawed and diluted in culture medium immediately before use. BZ purchased from Alfa Aesar (#J60378) was resuspended in DMSO to yield a 10 mM solution stored at −20°C. A 10 mM solution of carfilzomib in DMSO was purchased from ApexBio (#A1933) and stored at −20°C. BZ and carfilzomib were thawed and diluted in culture medium immediately before use. Thapsigargin (Tocris, #1138) and tunicamycin (Tocris, #3516) were resuspended in DMSO to yield a 10 mM solution stored at −20°C; and dithiothreitol (DTT, 1 M solution) was purchased from Invitrogen (#P2325) and stored at −20°C.

### MM cell line cultures and drug treatment

The MM.1S and KMS‐11 cell lines were from J.‐C.B. (Fayon *et al*, 2020). JIM3 cells were kindly provided by Dr. MacLennan (Birmingham Medical School, Birmingham, UK). All cell lines were tested negative for mycoplasma. Cells were cultured in RPMI 1640 medium supplemented with 10% fetal bovine serum (FBS, Dominique Dutcher #S1810‐500), 100 Units/ml penicillin + 100 µg/ml streptomycin (Gibco, #15140‐122). A BZ‐resistant version of the MM.1S cell line (MM.1S BzR) was generated by serial subculture in the presence of increasing doses of BZ, starting from 0.1 nM and doubling BZ concentration every 2 weeks until 0.8 nM, and then increasing BZ concentration by 0.1 nM every 2 weeks until 15 nM. Compared to parental cells, the growth rate of MM.1S BzR cells was not reduced and their BZ‐resistant phenotype was stable upon removal of BZ from culture medium. Exponentially growing MM.1S, JIM3, and KMS‐11 cells were plated in 96‐well plates at a density of 1.5 × 10^5^ cells/well (MM‐1S) or 10^5^ cells/well (JIM3 and KMS‐11). Cells were then treated with mycolactone and/or BZ at the indicated concentrations for 24, 48, or 72 h at 37°C.

### Lymphomas

Total bone marrow from 3‐ to 5‐week‐old female C57BL/6 mice was cultured and infected with a retrovirus encoding v‐abl kinase to generate immortalized pro‐B cell lines as previously described (Rosenberg *et al*, 1975; Lescale *et al*, 2016). v‐abl pro‐B cell lines were cultured in RPMI GlutaMAX^TM^ supplemented with 12% heat‐inactivated FBS, 10 mM Hepes, and 50 µM 2‐mercaptoethanol. Cells were treated with mycolactone and/or BZ as above.

### Flow cytometric analysis of MM cell lines

Following drug treatment, MM cells were pelleted and resuspended in flow cytometry buffer (1× PBS with 1% FBS) then incubated with human FcR blocking reagent (Miltenyi Biotec, # 130‐059‐901) before staining with diverse fluorescent dye‐conjugated monoclonal antibodies: anti‐CD126 (IL‐6Rα)/APC (#352806 Biolegend, dilution 1/50), anti‐CD38/BV421 (#303526 Biolegend, diluted 1/200), anti‐CD38/PE (#356604 Biolegend, diluted 1/200), anti‐CD138/APC (#130‐117‐395 Miltenyi Biotec, diluted 1/100), anti‐CD138/APC‐AF750 (#352316 Biolegend, diluted 1/100), anti‐CD40/FITC (#334306 Biolegend, diluted 1/25), and anti‐BCMA/PerCp‐Cy5.5 (#357510 Biolegend, diluted 1/25). Cells were stained for 15 min at 4°C, then washed and filtered before data acquisition by Cytoflex flow cytometer (Beckman Coulter). Data were analyzed with the FlowJo software (Tree Star, Ashland, OR). Mean fluorescence intensities (MFI) of isotype controls recommended by the Ab supplier were systematically subtracted to that of each stained sample. Cell viability was assessed by Annexin V exposure and PI incorporation using the FITC‐Annexin V/PI kit from Miltenyi Biotec (#130‐092‐052) following the manufacturer’s protocol. Live cells were characterized as Annexin V^‐^ PI^‐^, apoptotic cells as Annexin V^+^ PI^−^, and dead cells as Annexin V^+^ PI^+^ and Annexin V^−^ PI^+^ as shown in Appendix Fig S1.

### Detection of secreted immunoglobulin light chain by ELISA

Supernatants from MM cells cultures treated with mycolactone and/or BZ alone were collected after 24 h, and the concentration of free light chain was assessed using the SEBIA FLC lambda ELISA kit (SEBIA, 5101) according to the manufacturer’s recommendations.

### Western blot analyses

Following a 6 h treatment with mycolactone and/or BZ at the indicated concentrations, 2 × 10^6^ cells were harvested and solubilized at 10^8^ cells/ml for 15 min in ice‐cold lysis buffer containing 1% Nonidet P‐40, 1% n‐dodecyl‐{beta}‐D‐maltoside, 20 mM Tris‐HCl, pH 7.5, 150 mM NaCl, 1 mM MgCl2, and 1 mM EGTA in the presence of inhibitors of proteases and phosphatases (10 µg/ml leupeptin, 10 µg/ml aprotinin, 1 mM Pefabloc‐sc, 50 mM NaF, 10 mM Na4P2O7, and 1 mM NaVO4). For immunoblot analyses, loadings were normalized on the basis of the total amount of proteins per lane. Proteins were then separated by sodium dodecyl sulfate polyacrylamide gel electrophoresis (SDS‐PAGE) using NuPAGE Bis‐Tris gels (Thermo Fisher Scientific) and transferred on nitrocellulose membranes (iBlot 2^®^ gel transfer Stacks Nitrocellulose system from Invitrogen). Immune blotting was carried out with the primary antibodies anti‐ATF4 (Cell Signaling, #11815), anti‐GAPDH (Cell Signaling, #2118), and anti‐ATF6 (Biolegend, 853102) overnight at 4°C. After washing, the membranes were incubated with HRP‐conjugated anti‐rabbit, ‐mouse, or ‐rat IgG secondary antibodies (Santa Cruz, sc‐2004, Biolegend, 405405) for 45 min at room temperature. All antibodies were diluted 1/1,000 in PBS‐fat free milk (5% m/v). Detection of proteins was performed with the enhanced chemical luminescence (ECL) method using the ECL Prime Western Blotting Reagent, and image was acquired on an ImageQuant LAS 4000 Mini (GE Healthcare).

### Gene expression analyses

Following a 6 h treatment with mycolactone and/or BZ at the indicated concentrations, 2 × 10^6^ cells were harvested and lysed in Trizol (Qiagen). Chloroform was added to the trizol lysates, and the mix was then centrifuged for 15 min at 15,000 *g* and 4°C. After centrifugation, the aqueous phase was recovered and mixed with 1.5 volume of ethanol. Total mRNAs were then extracted using RNeasy Plus Mini Kit (Qiagen) according to the manufacturer’s procedure and reverse transcribed into cDNAs using High Capacity cDNA Reverse Transcription kit (BD Bioscience) from 1 µg total mRNA according to the manufacturer’s recommendations. The levels of transcription of the mRNAs coding for the genes of interest were assessed using SyberGreen (Power SYBR Green PCR Master Mix, applied biosystem ref: 4367659) and the following primers synthesized by Eurofins genomics: CHOP (forward), GCACCTCCCAGAGCCCTCACTCTCC; CHOP (reverse), GTCTACTCCAAGCCTTCCCCCTGCG; sXBP1 (forward), GGTCTGCTGAGTCCGCAGCAGG; sXBP1 (reverse), GGGCTTGGTATATATGTGG; XBP1total (forward), TGGCCGGGTCTGCTGAGTCCG; XBP1total (reverse), ATCCATGGGGAGATGTTCTGG; ATF4 (forward), CACCGCAACATGACCGAAAT; ATF4 (reverse), GACTGACCAACCCATCCACA; GADD34 (forward), GATGGCATGTATGGTGAGCG; GADD34 (reverse), GAGACAAGGCAGAAGTAGAG; BiP (forward), CGAGGAGGAGGACAAGAAGG; BiP (reverse), CACCTTGAACGGCAAGAACT. Quantitative PCR conditions used were as follows: 50°C 2 min, 95°C 10 min, 95°C 15 s (40 cycles), and 60°C for 1 min. The relative quantification was calculated by the 2^−ΔΔCT^ method and the 18S mRNA was used as endogenous control.

### RT‐PCR analysis

Total mRNA was extracted from MM.1S cells and cDNAs were synthetized as described above. PCR amplification was performed using GoTAQ Green Master Mix (Promega) and the following XBP‐1 primers: (forward) AAACAGAGTAGCAGCTCAGACTGC; (reverse) TCCTTCTGGGTAGACCTCTGGGAG. PCR products were digested using PstI (New England BioLabs) for 15 min at 37°C and separated on 2% agarose gel.

### Flow cytometric analysis of MM patient‐derived samples and PBMCs

Patient‐derived mononuclear cells were isolated from bone marrow aspirate by density gradient centrifugation with Pancoll separating solution (PAN‐Biotech, DE). 2 × 10^5^ cells were then plated in 96 wells plates in culture medium in the absence or the presence of mycolactone or BZ alone or in combination as described in the text for 18 h. Cells were then washed and stained with fluorescent‐conjugated anti‐CD38 and anti‐CD138 antibodies to gate plasma cells, in the presence of Annexin V‐FITC and PI for 15 min at room temperature. Cells were then acquired using a flow cytometer (Attune NXT, ThermoFisher) immediately after the staining and cell viability was assessed by analysis using FlowJo software (Tree Star, Ashland, OR). PBMCs were isolated from blood samples of healthy donors collected by the French Blood Establishment (EFS), by Pancoll density gradient centrifugation. 2 × 10^5^ cells were plated in 96‐well plates and treated with mycolactone, BZ, alone or in combination, for 18 h. Cells were then washed and stained with anti‐CD3/PerCPCy5.5 (#332771 BD Bioscience), anti‐CD19/FITC (#302206 Biologend), anti‐CD56/APC (#341027 BD Bioscience), anti‐CD16/PE‐CF594 (#562293 BD Bioscience), and anti‐CD11c/PE (#333149 BD Bioscience). Dead cells were identified using LIVE/DEAD Fixable Near IR Dead Cell Stain Kit (#L34975 Invitrogen). Cells were then acquired using a flow cytometer (Cytoflex, Beckman Coulter).

### Mice

Six‐ to eight‐week‐old female pathogen‐free C57BL/6J mice were purchased from Charles River Laboratories. The NOD. Cg‐*Prkdc^scid^ Il2rg^tm1Wjl^*/SzJ (NSG, stock number: 005557) mice were purchased from the Jackson laboratory and were used between 6 and 12 weeks of age. All mice were housed at animal facilities of the Institut Pasteur under specific pathogen‐free conditions with food and water *ad libitum*. Mouse experiments were validated by CETEA Ethics Committee number 0068 (Institut Pasteur, Paris, France, DAP170027) and received the approval of the French Ministry of Higher Education and Research. They were performed in compliance with national guidelines and regulations.

### Mouse experiments

*In vivo* toxicity assays were performed on 8‐week‐old C57BL/6J (B6) mice purchased from Charles River laboratories as follows. Mycolactone (0.3 mg/kg) and BZ (0.3 mg/kg) alone or in combination were diluted in NaCl solution (0.9% w/v) administrated every 3.5 days via the intraperitoneal route. The toxicity of the treatments was assessed by measuring the percentage of T and B cells in the blood sample collected in tubes containing EDTA (0.05 M) from the tail vein 5, 15, 20, and 25 days after the first injection. Red blood cells were lysed using red blood cells lysing buffer (SIGMA R7757). Leukocytes were stained with anti‐mouse CD3/PerCPcy5.5 (#45003182 eBioscience, diluted 1/100) and CD19/APC (#550992 BD Pharmingen, diluted 1/800) antibodies and fluorescence data were acquired by Cytoflex flow cytometer (Beckman Coulter). The percentages of T (CD3^+^) and B (CD19^+^) cells were determined using FlowJo software. Experiments assessing the effect of the treatments on the development of xenografted MM tumors were performed in 8‐ to 12‐week‐old NSG mice. 3 × 10^6^ human MM‐1S cells were subcutaneously injected in the right flank of the animals in 200 µl of PBS. After 24 h, mice were randomly assigned to four groups and 6 days later, each group was randomly assigned a treatment. Mice were injected by intraperitoneal route with mycolactone (0.3 mg/kg and/or BZ (0.3 mg/kg), in 200 µl NaCL (0.9% w/v), or vehicle as control, every 3.5 days. Tumor growth was assessed daily by unblinded measurement of tumor size with a digital caliper. Data are presented as the average of two perpendicular diameters (millimeters). Mice were sacrificed when the tumor diameter reached 20 mm or whenever animals showed clinical signs of pain according to ethical guidelines. No animal was excluded from our analyses.

### Synergy scores

Synergy between mycolactone and BZ was assessed with the combenefit software (Di Veroli *et al*, 2016), which calculates scores based on the Loewe additivity model using the dose response of each drug and *P*‐value ranges. Loewe synergy score is defined as S_Loewe_ = Y_obs_ ‐Y_Loewe_, where Y_obs_ is the observed effect of the combination and Y_Loewe_ is the theoretical effect of the combination. Therefore, a S_Loewe_ > 0 shows that drugs act in synergy. On the contrary, S_Loewe_ < 0 depicts an antagonist effect of the drugs. S_Loewe_ were plotted as heatmaps and statistical significance was analyzed by one‐sample Student’s *t*‐test.

### Statistical analyses

Other statistical treatments and graphical representations were performed with the Prism software (v8.4.3, GraphPad, La Jolla, CA) and values of *P* ≤ 0.05 were considered significant. Detailed information on the statistical test used and number of replicates is provided in figure legends. Positive controls were not considered in statistical analyses of qPCR data.

## Author contributions

AD, CC, LB, and VM performed the experiments. AD, EP, and GD performed the statistical analyses. LD provided *v‐abl* B‐cell clones and consultation regarding lymphomas. BA and J‐CB provided bone marrow aspirates from MM patients and guidance with their handling and flow cytometric analysis. AD, GD, and CD designed the experiments. Data interpretation and writing of the manuscript were performed by AD, GD, and CD.

## Supporting information



Appendix

Expanded View Figures PDF

Source Data for Expanded View

Source Data for Figure 3

Source Data for Figure 5

## Data Availability

This study includes no data deposited in external repositories.
