# Supplementary material for: The Sec61 translocon is a therapeutic vulnerability in multiple myeloma
Source: EMBO Mol Med. 2022 Jan 11;14(3):e14740. doi: 10.15252/emmm.202114740 (PMC8899908; doi:10.15252/emmm.202114740)

Appendix

Figure S1: Gating strategy for characterization of live, apoptotic and dead cells. Diagram illustrating our gating strategy in MM-1S cell treated for 72 h with mycolactone (50 nM) and then exposed to annexin V and PI. Areas corresponding to live (Annexin V^- PI^-), apoptotic (Annexin V^+ PI^-) and dead (PI^+) cells characterization are shown.

Figure S2: Gating strategy for identification of MM cells, lymphoid cells and monocytes/macrophages in bone marrow aspirates from MM patients. Diagram illustrating our gating strategy in Pancoll-isolated mononuclear cells from bone marrow aspirates of newly diagnosed patient #2 **(A)** or relapsed patient #7 **(B)**, following staining with anti-CD38 and anti-CD138 antibodies.

Figure S3: Gating strategy for identification of T cells, B cells, NK cells dendritic cells and monocytes/macrophages in PBMCs. Diagram illustrating our gating strategy in Pancoll-isolated PBMCs from one healthy donor, following staining with anti-CD3, anti-CD-19, anti-CD16, anti-CD56 and anti-CD11c antibodies.

Appendix Figure S1

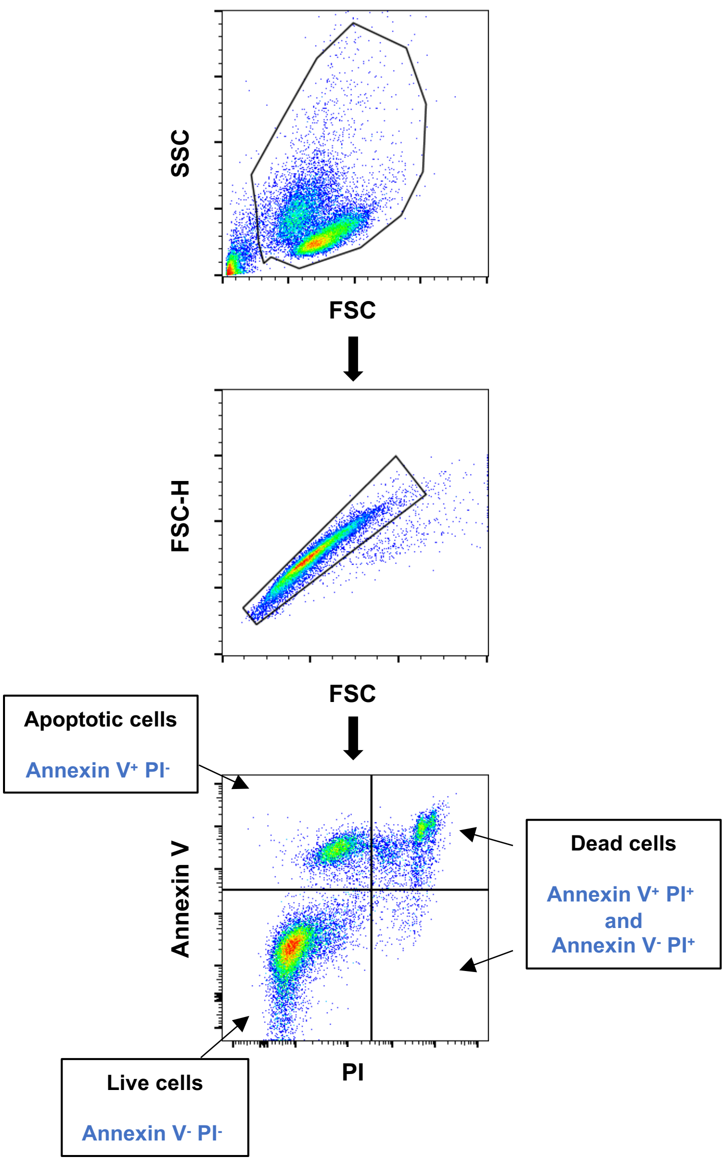

Appendix Figure S2

A. Diagnosed patient # 2

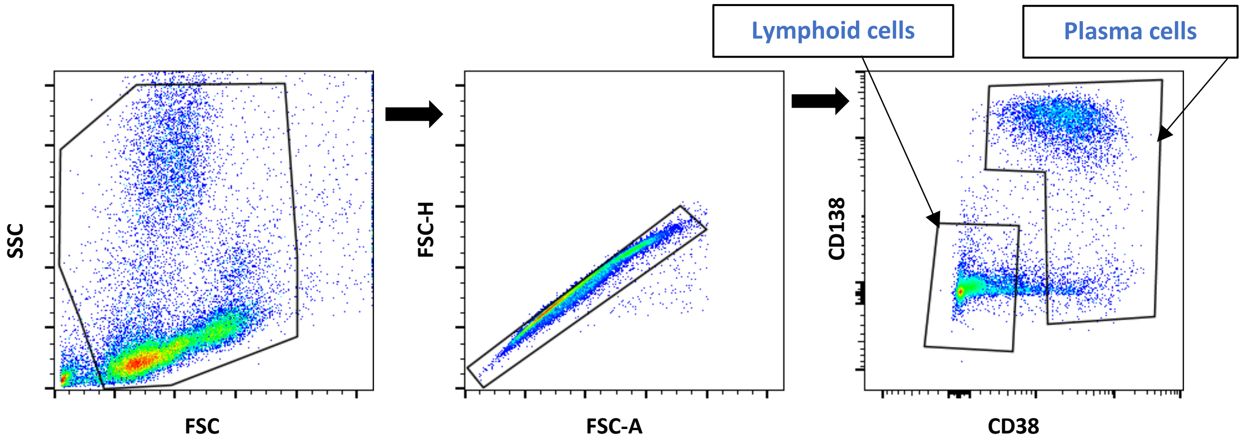

B. Relapsed patient # 7

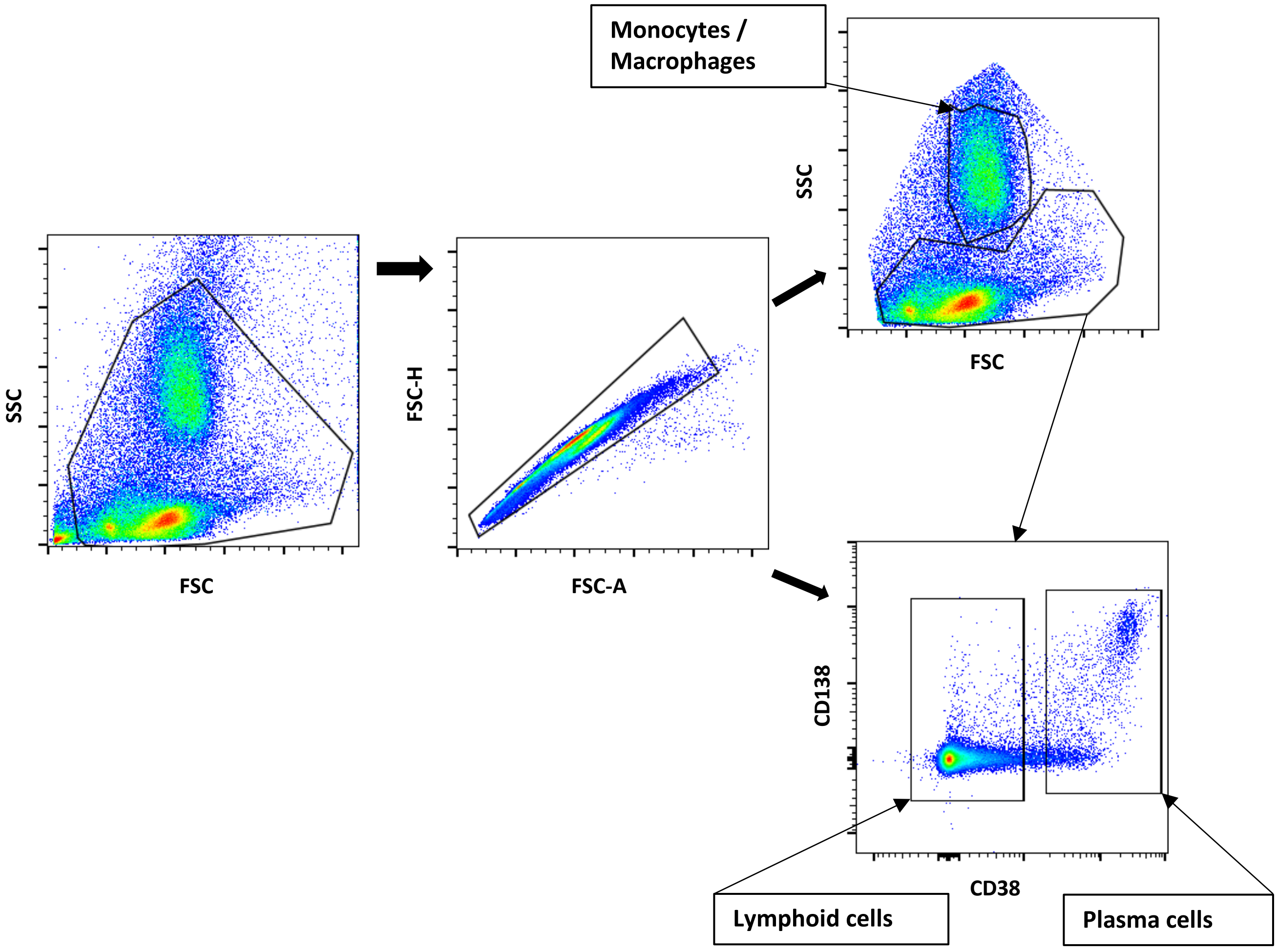

Appendix Figure S3

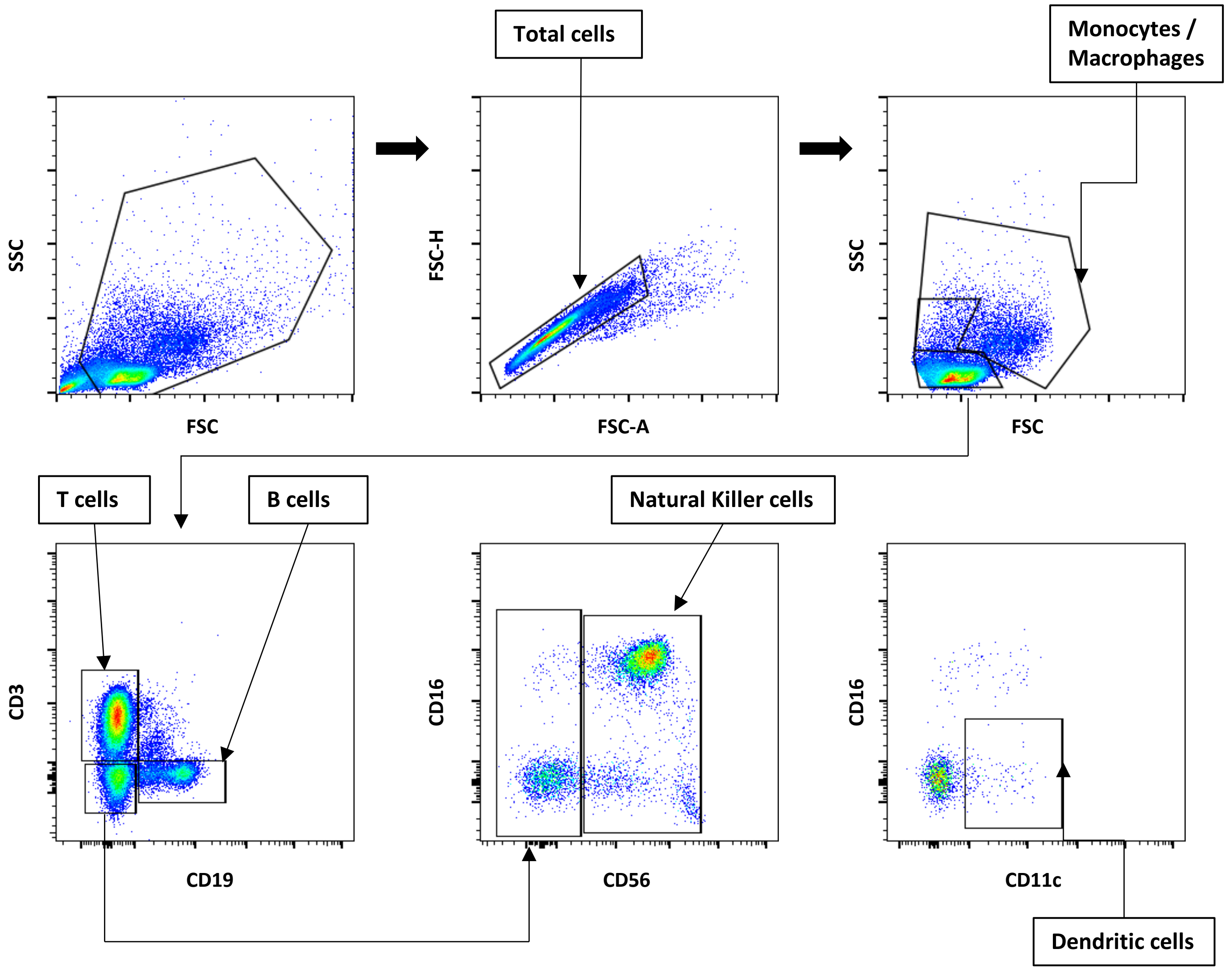

Supplement: Supplementary file 1 — Appendix [file EMMM-14-e14740-s006.pdf]
