# Supplementary material for: The Sec61 translocon is a therapeutic vulnerability in multiple myeloma
Source: EMBO Mol Med. 2022 Jan 11;14(3):e14740. doi: 10.15252/emmm.202114740 (PMC8899908; doi:10.15252/emmm.202114740)
Supplement: Supplementary file 2 — Expanded View Figures PDF [file EMMM-14-e14740-s002.pdf]

Expanded View Figures

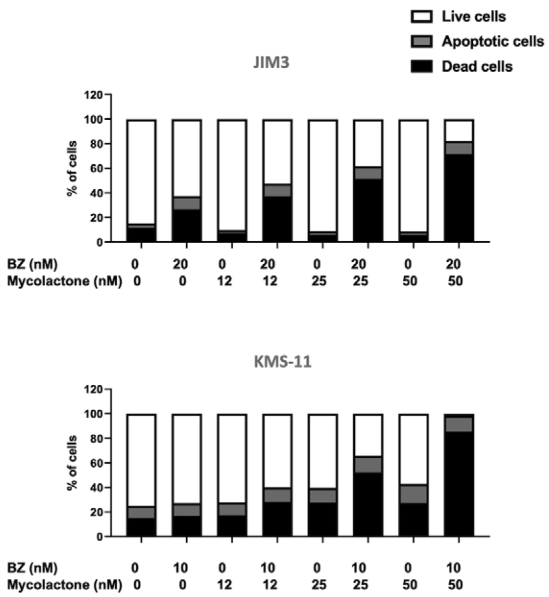

Figure EV1. Mycolactone potentiates the effect of BZ in JIM3 and KMS-11.

JIM3 and KMS-11 cells were treated with mycolactone and/or BZ at the indicated concentrations for 24 h. Data are Mean % of live, apoptotic, and dead cells from technical duplicates, gated as in Appendix Fig S1, relative to total cells. They are representative of two independent experiments with similar results.

 © 2022 The Authors

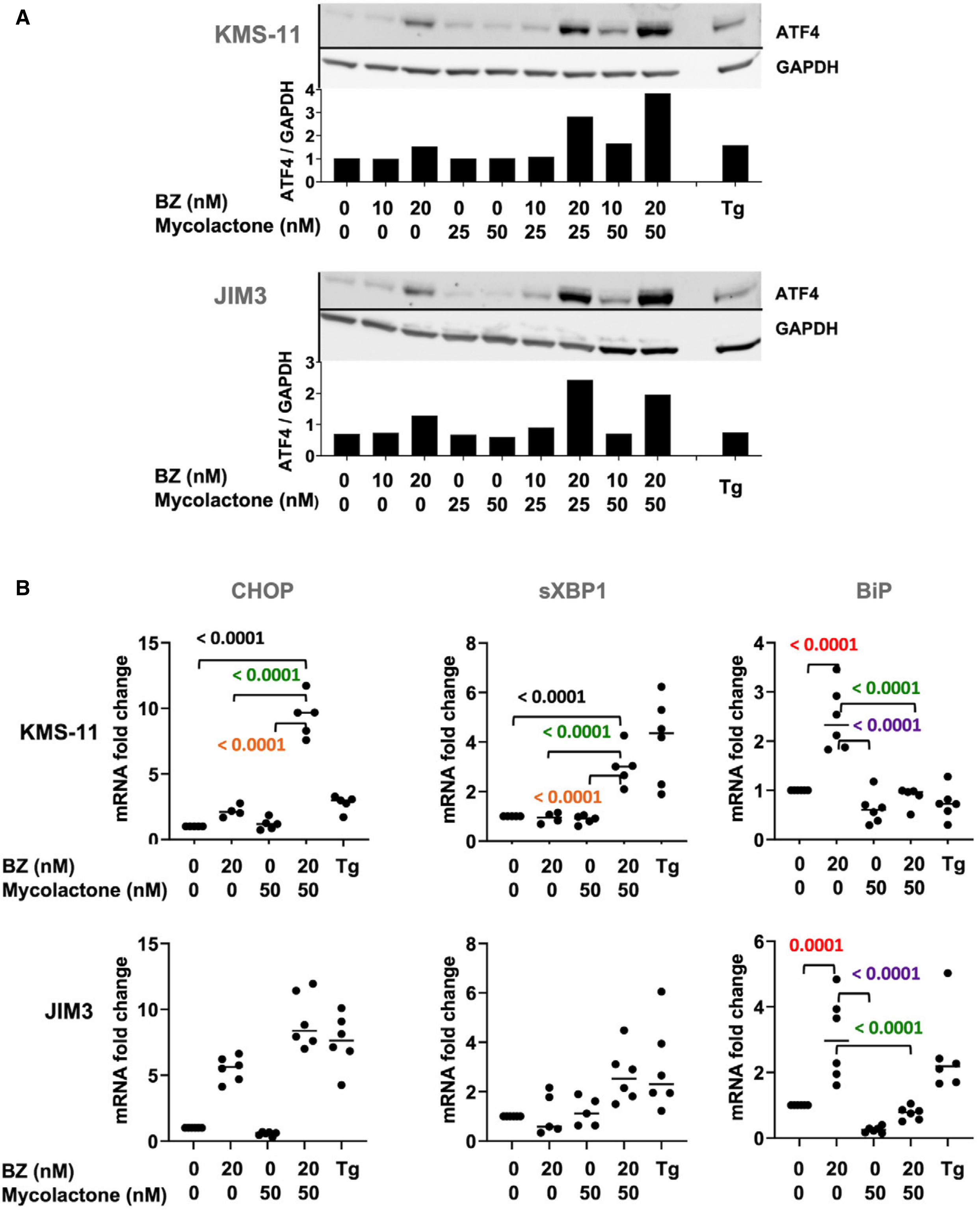
A
KMS-11
ATF4
GAPDH
ATF4 / GAPDH
BZ (nM) 0 10 20 0 0 10 20 10 20 Tg
Mycolactone (nM) 0 0 0 25 50 25 25 50 50
JIM3
ATF4
GAPDH
ATF4 / GAPDH
BZ (nM) 0 10 20 0 0 10 20 10 20 Tg
Mycolactone (nM) 0 0 0 25 50 25 25 50 50
B
CHOP
sXBP1
BiP
KMS-11
JIM3
mRNA fold change
< 0.0001
0.0001
BZ (nM) 0 20 0 20 Tg
Mycolactone (nM) 0 0 50 50

Figure EV2.

Figure EV2. The mycolactone–BZ combination activates pro-apoptotic UPR in KMS-11 and JIM3 cells.

A KMS-11 and JIM3 cells were treated with mycolactone and/or BZ, or vehicle as control, for 6 h. ATF4 protein levels were assessed in cell lysates by Western blot (top panel) and quantified relative to GADPH levels (lower panel). Data are representative of two independent experiments with similar results.

B CHOP, sXBP1, and BiP mRNA levels were quantified by qPCR in the two cell lines treated as in (A). sXBP1 mRNA levels were normalized to total (spliced + unspliced) XBP1 mRNA level. Data are Mean RNA fold changes ($2^{-\Delta\Delta CT}$) ± SD, relative to untreated controls. $N \geq 5$ (cumulative data of two independent experiments with technical duplicates and triplicates), pairwise compared by nested one-Way ANOVA with Tukey's multiple-comparison test, exact *P*-values indicated. Thapsigargin (Tg, 2 µM, 6 h) was used as a positive control.

Source data are available online for this figure.

© 2022 The Authors

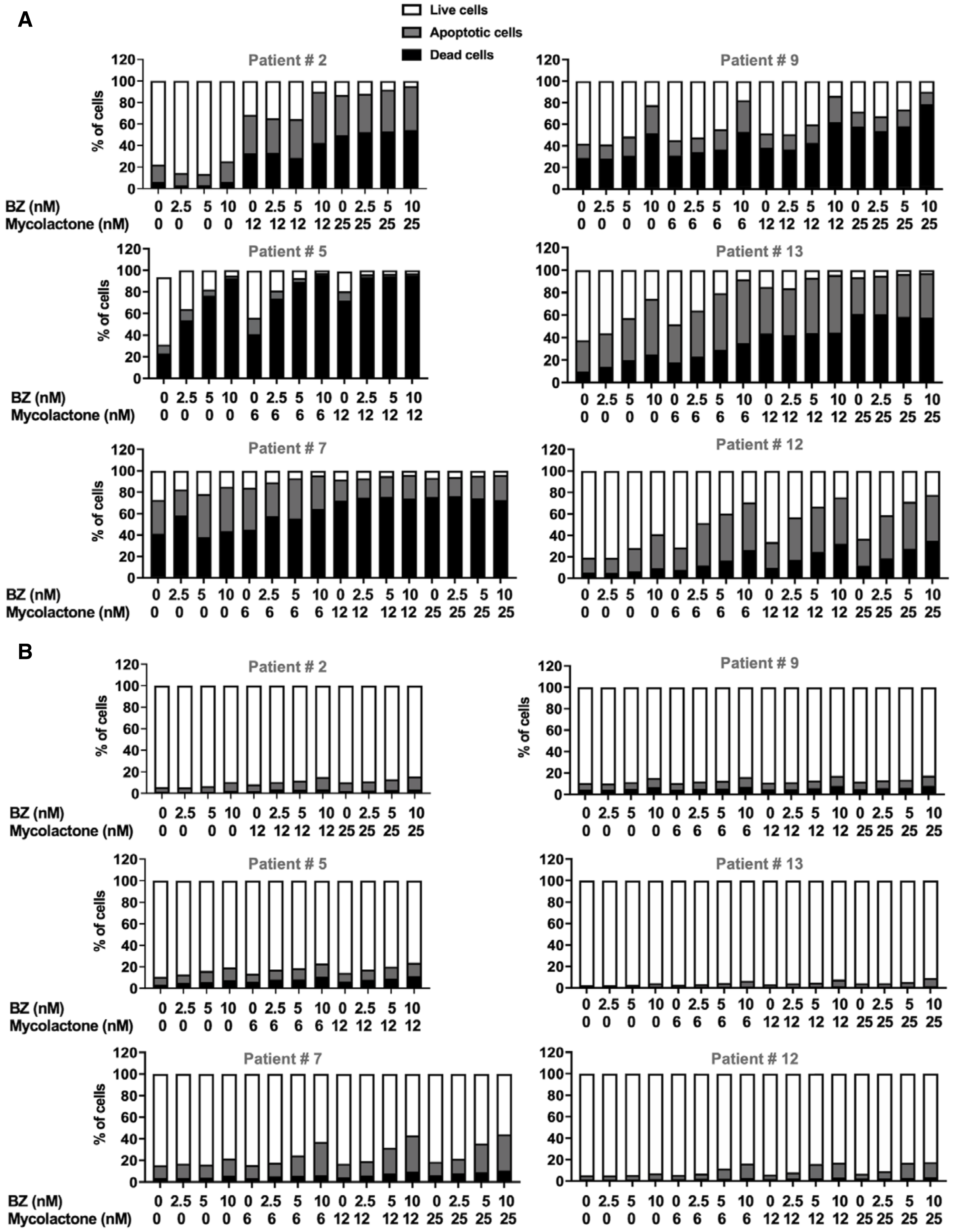

Figure EV3. Differential toxicity of mycolactone–BZ combination in MM cells and non-cancerous lymphoid cells in bone marrow aspirates.

A, B Incidences of live, apoptotic, and dead cells within the MM cell (A) and the non-cancerous lymphoid cell (B) subsets are compared in newly diagnosed (#2, #5, #9, and #13) or relapsed (#7 and #12) MM patients after an 18 h treatment with mycolactone and/or BZ. MM cells (A) and non-cancerous lymphoid cells (B) were identified with the gating strategy depicted in Appendix Fig S2. Data are Mean % of live, apoptotic, and dead cells from technical duplicates, relative to total cells.

© 2022 The Authors

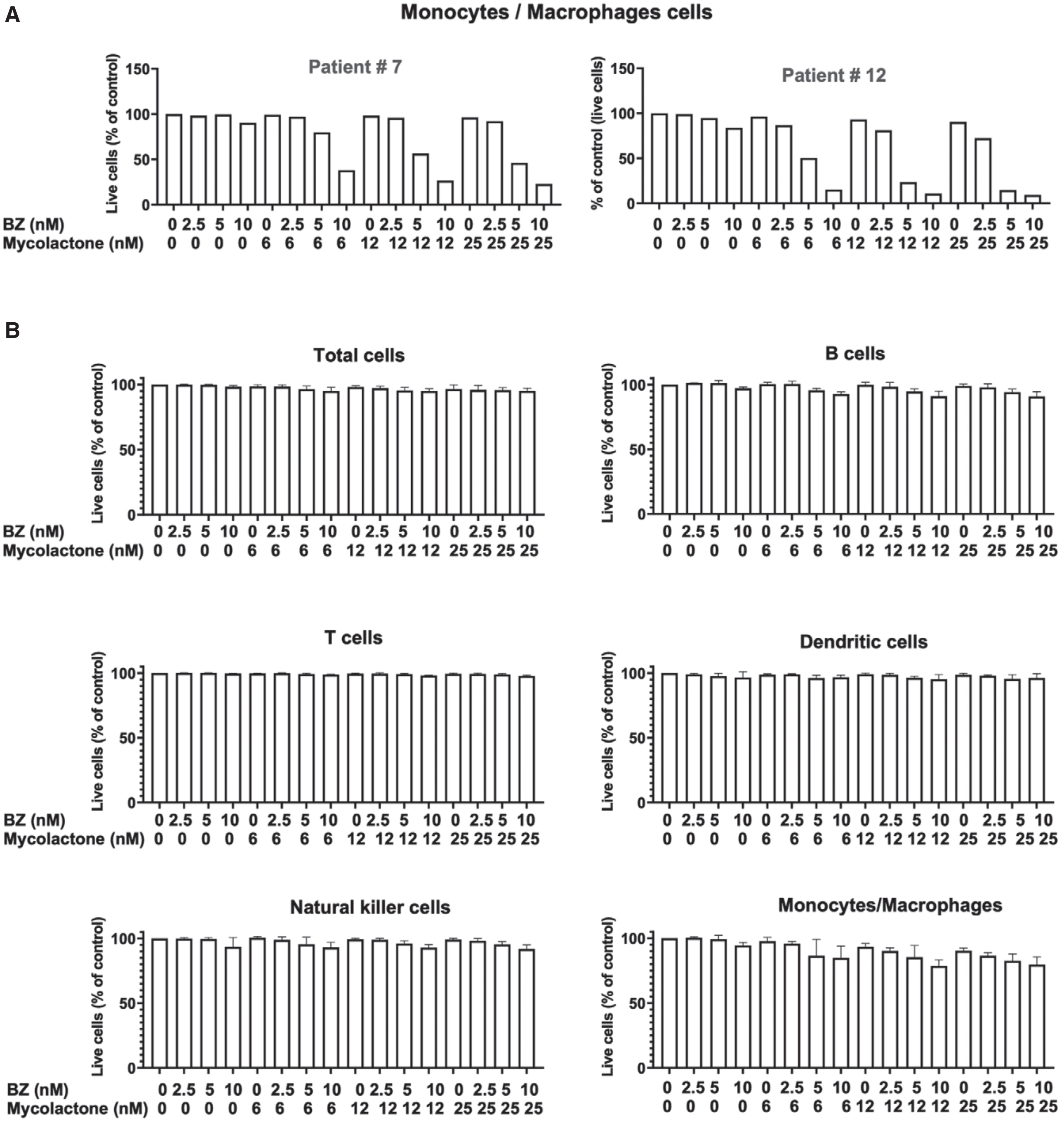

Figure EV4. Toxicity of the mycolactone–BZ combination in PBMCs and bone marrow monocytes/macrophages.

A The proportion of live (Annexin V$^-$ PI$^-$) cells within the monocyte/macrophage subset of mononuclear cells, after a 18 h treatment with mycolactone and/or BZ is shown for the two relapsed patients. Mononuclear cells from bone marrow aspirates were treated as in Fig 6. The monocyte/macrophage subset was identified using the gating strategy depicted in Appendix Fig S2. Data are Mean % of live cells, relative to vehicle controls.

B PBMCs from healthy donors were treated with mycolactone and BZ, alone or in combinations, at the indicated concentration for 18 h. Cells were then labelled with fluorophore-conjugated anti-CD3, anti-CD19, and anti-CD11c. Total cells, B cells, T cells, dendritic cells, NK cells, and monocytes/macrophages were identified by flow cytometry analysis, using the strategy depicted in Appendix Fig S3. Data are Mean % ± SD of live cells, relative to vehicle controls. $N = 6$, cumulative data from three donors with technical duplicates.

 © 2022 The Authors
