# Supplementary figures and images for: The Sec61 translocon is a therapeutic vulnerability in multiple myeloma
Source: EMBO Mol Med. 2022 Jan 11;14(3):e14740. doi: 10.15252/emmm.202114740 (PMC8899908; doi:10.15252/emmm.202114740)

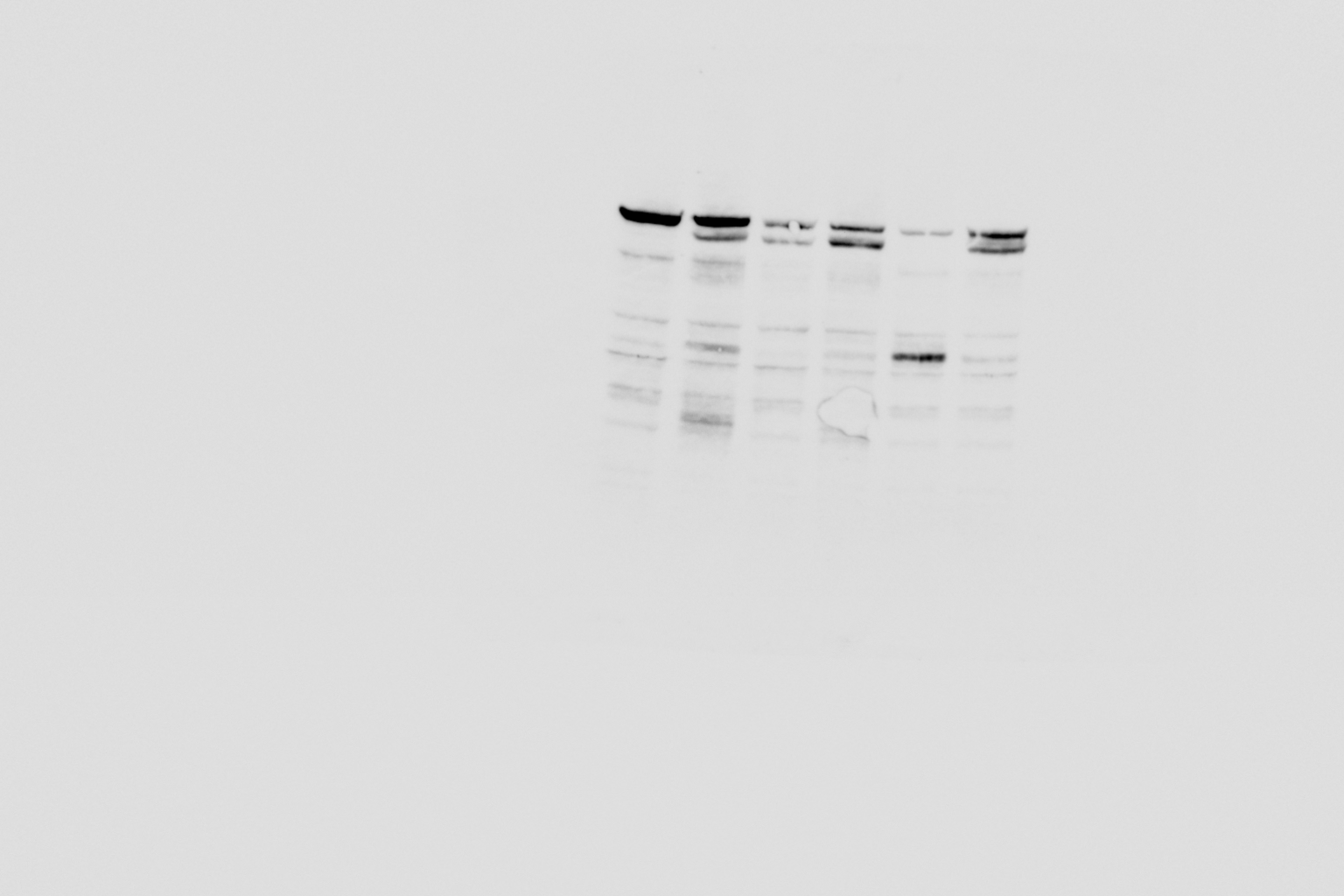

Supplement: Supplementary file 4 — Source Data for Figure 3 [file EMMM-14-e14740-s003.zip › Figure_3/Fig3E_ATF6_MM1S.tif]

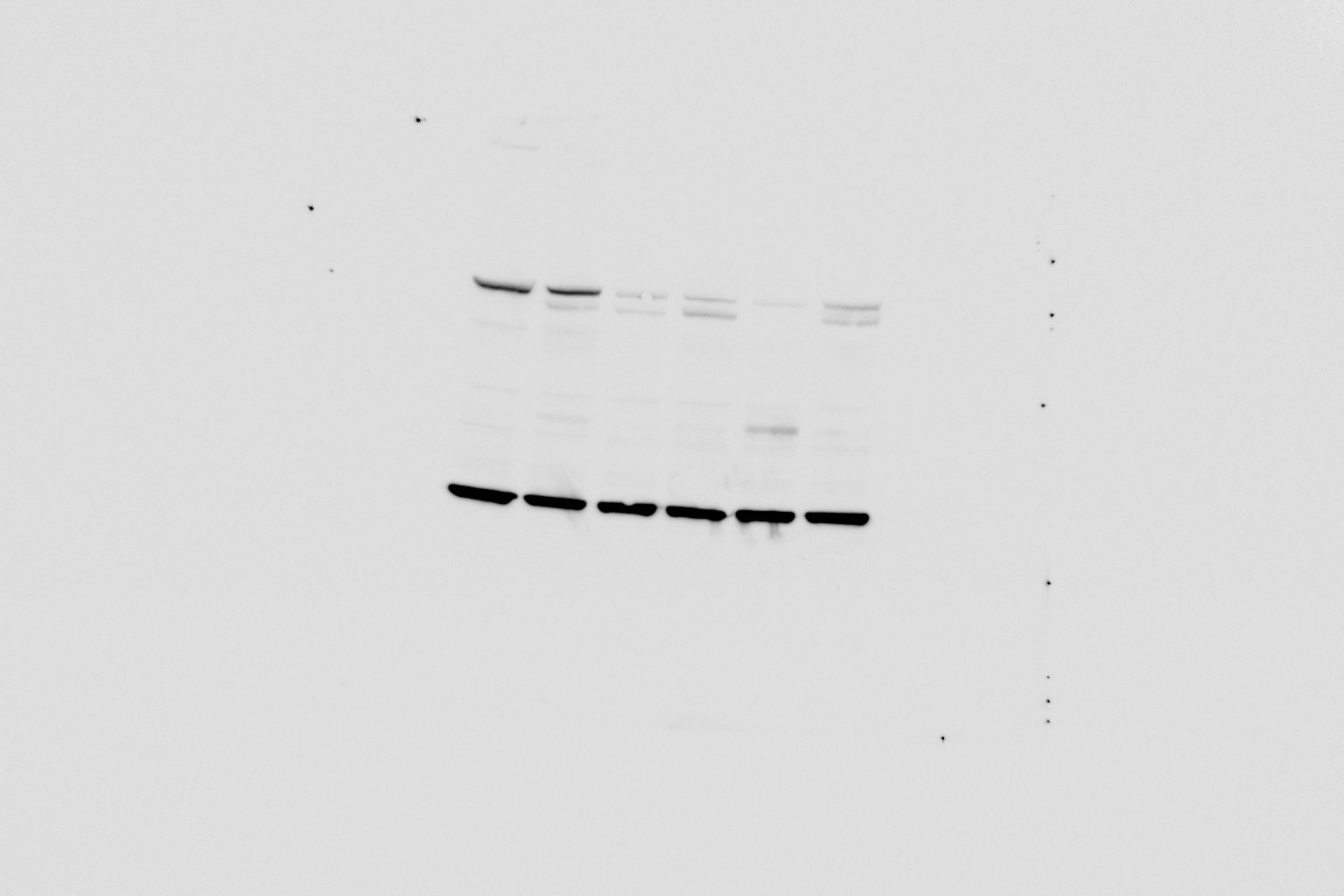

Supplement: Supplementary file 4 — Source Data for Figure 3 [file EMMM-14-e14740-s003.zip › Figure_3/Fig3E_GAPDH_MM1S.tif]
