# Supplementary material for: The Sec61 translocon is a therapeutic vulnerability in multiple myeloma
Source: EMBO Mol Med. 2022 Jan 11;14(3):e14740. doi: 10.15252/emmm.202114740 (PMC8899908; doi:10.15252/emmm.202114740)
Supplement: Supplementary file 6 [file EMMM-14-e14740-s005.pdf]

EMBO Molecular Medicine

The Sec61 translocon is a therapeutic vulnerability in Multiple Myeloma

Antoine Domenger, Caroline Choisy, Ludivine Baron, Veronique Mayau, Emeline Perthame, Ludovic Deriano, Bertrand Arnulf, Jean-Christophe Bories, Gilles Dadaglio, and Caroline Demangel
DOI: 10.15252/emmm.202114740

Corresponding authors: Caroline Demangel (demangel@pasteur.fr) , Gilles Dadaglio (gilles.dadaglio@pasteur.fr)

Review Timeline:

Submission Date:	18th Jun 21
Editorial Decision:	22nd Jul 21
Appeal Received:	23rd Jul 21
Editorial Decision:	3rd Aug 21
Revision Received:	18th Nov 21
Editorial Decision:	2nd Dec 21
Revision Received:	21st Dec 21
Accepted:	22nd Dec 21

Editor: Lise Roth

Transaction Report:

(Note: With the exception of the correction of typographical or spelling errors that could be a source of ambiguity, letters and reports are not edited. Depending on transfer agreements, referee reports obtained elsewhere may or may not be included in this compilation. Referee reports are anonymous unless the Referee chooses to sign their reports.)

22nd Jul 2021

Decision on your manuscript EMM-2021-14740

Dear Dr. Demangel,

Thank you for the submission of your manuscript to EMBO Molecular Medicine, and please accept my apologies for the delay in getting back to you. We have now received the reports from the three referees who agreed to review your manuscript.

As you will see, while the referees all mention the potential interest of the study, they also raise concerns regarding the lack of mechanistic understanding, the limited conceptual novelty, and the uncertain clinical relevance due to the potential toxicity of the treatment. Given these considerations, I am afraid that we cannot offer to consider the manuscript further.

While we cannot pursue this manuscript further, we encourage you to transfer your study to our not-for-profit open-access sister journal, Life Science Alliance (LSA). We shared your manuscript and the accompanying reviews with LSA Executive Editor, Eric Sawey, who is interested in these findings, and would like to publish this manuscript at LSA pending the following revisions:

- Address Reviewer 1's two specific points
- Address Reviewer 2's comments via Discussion. Comment #4 can be excluded, unless you readily have additional data to provide
- Address Reviewer 3's points via Discussion and in your Response to Reviewers. The only additional experimental data required is in response to the request to compare mycolactone toxicity to that of common ER stress inducers, such as Tg.

We encourage you to use the link below to transfer your manuscript to LSA. You do not need to revise the manuscript before transferring it to LSA. Once you transfer, Dr. Sawey will email you an invitation to revise and resubmit, listing the same revision requests as mentioned above. Please feel free to reach out at e.sawey@life-science-alliance.org if you have any questions about the LSA journal, the transfer process or the revisions requested.

I am very sorry to disappoint you on this occasion and I hope you will view the possibility of a transfer favorably.

Yours sincerely,

Lise Roth

Lise Roth
Editor
EMBO Molecular Medicine

Referee #1 (Comments on Novelty/Model System for Author):

The paper is interesting but not incredibly imaginative.

Referee #1 (Remarks for Author):

The paper by Domenger et al presents the case for an effective combination between BZ and Mycolactone in causing apoptosis of MM cells and relenting the course of an experimental model of MM in the mouse.

The results are clear and well presented but overall following a linear logic based in part on the group's previous reports. IN this regard, the present paper is essentially limited to the analysis of ATF4 as a mediator of apoptosis downstream of the PERK pathway.

The limitation of the study include:

1. A limited analysis of the IRA1 pathway. At the very least gel banding to show XBP1 splicing should be performed in addition to the RT-qPCR.

2. the phosphorylation of eIF2 alpha is worth exploring in connection with their group report in Cell Death and Disease (2018) that Mycolactone induces alone an integrated stress response. What is the effect of the combo treatment on this aspect of the UPR/ISR?

Apart from these considerations, the paper represents a clear opportunity to develop new combo treatments for MM.

Referee #2 (Comments on Novelty/Model System for Author):

Antoine Domenger et al reported that an Sec61 translocon inhibitor, mytolactone, induces MM cell death through ER stress and apoptosis, and has synergistic anti-MM effect with bortezomib. They provided in vitro, in vivo and clinical specimens to verify this phenotype. Generally this study is interesting, and a nM level anti-MM dosage has potential translational merit. However, this study lacks of molecular mechanistic investigations, and since Dr. Caroline Demangel has reported that mycolactone activates an atypical endoplasmic reticulum stress response(Mol Cell Proteomics, 2018. doi: 10.1074/mcp.RA118.000824.), and proteasome inhibitor Bortezomib also kills MM cells through inducing ER stress, thus this synergistic effect is reasonable. The following issues should be addressed:

1. To target sec61 in MM cells, we must know the significance of sec61 translocon in MM cells, Desirée Schubert et al reported that SEC61A1-V85D triggered the terminal unfolded protein response in MM cells, and it disturbs the cellular calcium homeostasis in HeLa cells, thus they found it plays role in PC deficiency and pathogenesis of antibody deficiencies. What is the role and significance of sec61 in the clinic should be investigated.

2. Abstract: Notably, this synergy was selective of cancer cells and extended to B cell acute lymphoblastic leukemia. Why the authors focus on MM but not on ALL?

3. Fig 1A, it seems mycolactone is more effective to JIM-3 and KMS-11 cells. Since these two cells are t(4;14) positive and express high level of methyltransferase NSD2, is it possible that mycolactone kills MM is NSD2 or H3K36me2 dependent?

4. All three pathways of ER stress should be investigated.

5. Fig 6A, there is discrepancy in the figure legend and figure labels. Figure legend has 5 groups, but figure label only shows 4.

Referee #3 (Comments on Novelty/Model System for Author):

This work pursues combination therapy of MM, a tumor that is well known to be susceptible to ER stress and whose standard therapy uses inhibition of proteasomes in order to increase misfolded protein stress. In general, the work is well done, uses three different established MM lines as well as ex vivo tumor-derived cells from new patients. The data about mycolactone as a cytotoxic agent is robust, detailed and interesting, the novelty being that mycolactone is a specific inhibitor of the sec61 translocon in the ER membrane, a different type of target than is usually employed in cancer therapy. The only additional aspect missing, to my mind, is comparison of mycolactone toxicity to that of common ER stress inducers, such as Tg (used as control in Figures 3-4), because presumably the relevant concentration would be considerably subtoxic when used alone and should induce much less ER stress than the Tg treatment.

The data in the paper shows that in two of the MM lines, combinations of mycolactone and BZ induce more ATF4 expression than the Tg control, which is an interesting observation that is and worth an explanation, given that the purpose is to convince that combination therapy is worth pursuing. Other strengths of the work are the inclusion of ex vivo human cells, testing tumor progression in a mouse model, and the extension of the mycolactone findings to B-ALL, which are a much more common clinical problem than MM.

One shortcoming of the work is the absence of BZ-resistant cells that would represent the population which most critically would benefit from combination therapy. The work uses the KMS-11 line, which is relatively more resistant to BZ than the other cell lines (Fig. 2A), but among the patient-derived cells there are none that are models of BZ escape mutants.

Importantly, the authors demonstrate the drug synergy in ex vivo patient cells (Figure 5) and show that the combination slows down tumor growth more than either BZ or mycolactone alone (Figure 6). I accept the conclusion, but note that the relatively BZ-sensitive line MM.1s was used in this setup, so it remains to be seen if more resistant cells, or even BZ-refractive cells exhibit the

same sensitivity.

Figure S5: I find the layout of part B, the non-cancerous cells, somewhat misleading. The design of this figure is different from all the others, where the entire population is accounted for. For example, in the bottom panel, the viability in most conditions is less than 100, yet there are no details about cells that are not presented in the diagrams. This becomes important when one tries to interpret the effects of mycolactone on non-cancerous cells. The authors claim no effect on non-cancerous cells (p.8), even though the target is present in them, and this point is important for assessing the selectivity of the drug.

A major point that I am not sure about is the level of novelty in the results presented. The inhibitor that is used by the authors is new but well-characterized in a number of published papers and it is not surprising that using it provides a major inhibition of secretory processes. However, the inhibitor does not target a protein or a process that is expressed differentially in MM cells or other cancers; It inhibits secretory protein production in all cells. Therefore, using the combination of Mycolactone and proteasome inhibitors is expected to cause general cytotoxicity and tissue damage, with the novel added parameter of lower doses than would be required if using each drug separately. This consideration limits, in my mind, the novelty of the data in this manuscript and its usefulness in clinical treatment. Assuming that EMBO Molecular Medicine has a high standard of quality, it is devateable whether the current work meets such standard.

========================

As a service to authors, EMBO provides authors with the possibility to transfer a manuscript that one journal cannot offer to publish to another EMBO publication. The full manuscript and if applicable, reviewers reports are automatically sent to the receiving journal to allow for fast handling and a prompt decision on your manuscript. For more details of this service, and to transfer your manuscript to another EMBO title please click on Link Not Available

Please do not share this URL as it will give anyone who clicks it access to your account.

Dear Lise,

Thank you for handling our manuscript. I would like to inform you of key new data directly addressing the reviewers' major concerns that we hope will encourage you to reverse your decision.

Since submission we have generated a sub-line of the relatively BZ-sensitive MM.1S cell line that has stable and >15x increased resistance to BZ. Importantly, it remains as sensitive to Mycolactone as the parental cell line, and the Mycolactone-BZ synergy is maintained. The BZ-resistant version of MM1-S thus provides the model of BZ escape mutants requested by Rev#3. These new findings eliminate any uncertainties regarding the clinical relevance of Sec61 inhibitor-based treatments.

Moreover, we would like to refute Rev#3's claim that mycolactone targets a ubiquitous process that is likely to cause global toxicity. Even though Sec61 is expressed by all cells we provide robust in vitro and in vivo evidence that its inhibition selectively affects the viability of highly secretory MM cells, and that there is a therapeutic window for Sec61 inhibitors, alone and combined with proteasome inhibitors. This is clearly recognized by Rev#2, who states that the 'nM level anti-MM dosage has potential translational merit'.

We can easily meet all raised minor criticisms by (1) providing a more detailed analysis of the treatment impact on the different arms of the UPR/ISR pathways, (2) comparing the toxicity of mycolactone (and combination with BZ) to that of the typical ER stressor Tg, and (3) answering Rev#2's open questions in the Discussion.

Since all Reviewers found our study 'interesting', and Rev#1 even qualified it as 'a clear opportunity to develop combo treatments for MM', we believe that the above clarifications and the additional new data we can provide should make it acceptable for further consideration by EMBO Mol. Med. I hope you agree.

Caroline

3rd Aug 2021

Dear Caroline,

Thank you for your email asking us to reconsider our decision on your manuscript based on new data that could potentially address the reviewers' concerns. I have communicated your letter to the referees, and I have now received feedback from two of them.

They both agree that the addition of a new resistant cell line would address some of their main issues and would thus be willing to review a revised manuscript. They further mention that it would be important to:
- Discuss the possible mechanisms by which a drug to a ubiquitous target like sec61, which by itself has not cytotoxic effect, can synergize with another drug to kill MM cells more effectively,
- Discuss the potential effects of chronic treatment with mycolactone in light of previous studies (i.e. PMID: 19934005 and 29320578)

If you feel you can satisfactorily address these points as well as those listed in the initial reports, you may wish to submit a revised version of your manuscript. Please attach a covering letter giving details of the way in which you have handled each of the points raised by the referees. A revised manuscript will once again be subject to review, and we cannot guarantee at this stage that the eventual outcome will be favorable.

Revised manuscripts should be submitted within three months of a request for revision; they will otherwise be treated as new submissions, except under exceptional circumstances in which a short extension is obtained from the editor.

When submitting your revised manuscript, please carefully review the instructions that follow below. We perform an initial quality control of all revised manuscripts before re-review; failure to include requested items will delay the evaluation of your revision.

We require:

1) A .docx formatted version of the manuscript text (including legends for main figures, EV figures and tables). Please make sure that the changes are highlighted to be clearly visible.

2) Individual production quality figure files as .eps, .tif, .jpg (one file per figure). For guidance, download the 'Figure Guide PDF' (https://www.embopress.org/page/journal/17574684/authorguide#figureformat).

3) A .docx formatted letter INCLUDING the reviewers' reports and your detailed point-by-point responses to their comments. As part of the EMBO Press transparent editorial process, the point-by-point response is part of the Review Process File (RPF), which will be published alongside your paper.

4) A complete author checklist, which you can download from our author guidelines (https://www.embopress.org/page/journal/17574684/authorguide#submissionofrevisions). Please insert information in the checklist that is also reflected in the manuscript. The completed author checklist will also be part of the RPF.

5) It is mandatory to include a 'Data Availability' section after the Materials and Methods. Before submitting your revision, primary datasets produced in this study need to be deposited in an appropriate public database, and the accession numbers and database listed under 'Data Availability'. Please remember to provide a reviewer password if the datasets are not yet public (see https://www.embopress.org/page/journal/17574684/authorguide#dataavailability).

In case you have no data that requires deposition in a public database, please state so in this section. Note that the Data Availability Section is restricted to new primary data that are part of this study.

6) For data quantification: please specify the name of the statistical test used to generate error bars and P values, the number (n) of independent experiments (specify technical or biological replicates) underlying each data point and the test used to calculate p-values in each figure legend. The figure legends should contain a basic description of n, P and the test applied. Graphs must include a description of the bars and the error bars (s.d., s.e.m.).

7) We would also encourage you to include the source data for figure panels that show essential data. Numerical data should be provided as individual .xls or .csv files (including a tab describing the data). For blots or microscopy, uncropped images should be submitted (using a zip archive if multiple images need to be supplied for one panel). Additional information on source data and instruction on how to label the files are available at
.

8) Our journal encourages inclusion of *data citations in the reference list* to directly cite datasets that were re-used and obtained from public databases. Data citations in the article text are distinct from normal bibliographical citations and should directly link to the database records from which the data can be accessed. In the main text, data citations are formatted as follows: "Data ref: Smith et al, 2001" or "Data ref: NCBI Sequence Read Archive PRJNA342805, 2017". In the Reference list, data citations must be labeled with "[DATASET]". A data reference must provide the database name, accession number/identifiers and a resolvable link to the landing page from which the data can be accessed at the end of the reference. Further instructions are available at .

9) We replaced Supplementary Information with Expanded View (EV) Figures and Tables that are collapsible/expandable online. A maximum of 5 EV Figures can be typeset. EV Figures should be cited as 'Figure EV1, Figure EV2" etc... in the text and their respective legends should be included in the main text after the legends of regular figures.

- For the figures that you do NOT wish to display as Expanded View figures, they should be bundled together with their legends in a single PDF file called *Appendix*, which should start with a short Table of Content. Appendix figures should be referred to in the main text as: "Appendix Figure S1, Appendix Figure S2" etc.

- Additional Tables/Datasets should be labeled and referred to as Table EV1, Dataset EV1, etc. Legends have to be provided in a separate tab in case of .xls files. Alternatively, the legend can be supplied as a separate text file (README) and zipped together with the Table/Dataset file.

See detailed instructions here:
.

10) For more information: There is space at the end of each article to list relevant web links for further consultation by our readers. Could you identify some relevant ones and provide such information as well? Some examples are patient associations, relevant databases, OMIM/proteins/genes links, author's websites, etc...

11) Every published paper now includes a 'Synopsis' to further enhance discoverability. Synopses are displayed on the journal webpage and are freely accessible to all readers. They include a short stand first (maximum of 300 characters, including space) as well as 2-5 one-sentences bullet points that summarizes the paper. Please write the bullet points to summarize the key NEW findings. They should be designed to be complementary to the abstract - i.e. not repeat the same text. We encourage inclusion of key acronyms and quantitative information (maximum of 30 words / bullet point). Please use the passive voice. Please attach these in a separate file or send them by email, we will incorporate them accordingly.

Please also suggest a striking image or visual abstract to illustrate your article as a PNG file 550 px wide x 300-600 px high.

12) As part of the EMBO Publications transparent editorial process initiative (see our Editorial at http://embomolmed.embopress.org/content/2/9/329), EMBO Molecular Medicine will publish online a Review Process File (RPF) to accompany accepted manuscripts.
In the event of acceptance, this file will be published in conjunction with your paper and will include the anonymous referee reports, your point-by-point response and all pertinent correspondence relating to the manuscript. Let us know whether you agree with the publication of the RPF and as here, if you want to remove or not any figures from it prior to publication.
Please note that the Authors checklist will be published at the end of the RPF.

EMBO Molecular Medicine has a "scooping protection" policy, whereby similar findings that are published by others during review or revision are not a criterion for rejection. Should you decide to submit a revised version, I do ask that you get in touch after three months if you have not completed it, to update us on the status.

I look forward to seeing a revised form of your manuscript as soon as possible. Use this link to login to the manuscript system and submit your revision: Link Not Available

With my best wishes,

Lise

Lise Roth, Ph.D
Editor
EMBO Molecular Medicine

Referee #1

The results are clear and well presented but overall following a linear logic based in part on the group's previous reports. IN this regard, the present paper is essentially limited to the analysis of ATF4 as a mediator of apoptosis downstream of the PERK pathway.

1. A limited analysis of the IRE1 pathway. At the very least gel banding to show XBP1 splicing should be performed in addition to the RT-qPCR.

In addition to our previous analysis of spliced XBP1 by qPCR, activation of the IRE1 pathway in MM.1S cells is now analyzed by gel electrophoresis analysis of RT-PCR amplified/Pst1 digested products of XBP1 mRNAs. Both analyses demonstrate a significant activation of XBP1 splicing by the mycolactone/BZ combination, compared to single drugs (Fig. 3F). Our previous analysis of drug impact on XBP1 splicing in the 2 other MM cell lines (KMS-11 and JIM3) is now shown in Fig. EV2. The revised version of our manuscript also includes this analysis in the newly generated BZ-resistant version of MM.1S (see our answer to Referee #3 and Fig. 4).

We also performed a more detailed analysis of mycolactone/BZ impact on the 2 other, PERK and ATF6-mediated pathways of ER stress in MM.1S cells (see our answers to point 2 below and point 4 of Referee #2). Corresponding data are grouped in Figure 3. In addition to KMS-11 and JIM3 (Fig. EV2), induction of ATF4/CHOP signaling was examined in the BZ-resistant version of MM.1S cells (new Fig. 4). In all studies MM cells, analysis of ER stress was completed by a qPCR analysis of the UPR regulator BiP (Fig. 3, 4 and EV2).

2. the phosphorylation of eIF2 alpha is worth exploring in connection with their group report in Cell Death and Disease (2018) that Mycolactone induces alone an integrated stress response. What is the effect of the combo treatment on this aspect of the UPR/ISR?

Induction of the ISR by mycolactone was reported by the group of Rachel E Simmonds, in a study using mouse embryonic fibroblasts and HeLa cells (*1*). The authors found that mycolactone activates the PERK/eIF2α/ATF4/CHOP pathway located at the intersection between the UPR and the ISR, without apparent activation of UPR-specific signals such as XBP1 splicing and ATF6 cleavage. With regard to XBP1 splicing, this contrasts with previous work from our lab using a dendritic cell line (*2*) and the present study using MM cell lines, suggesting that mycolactone-driven activation of the IRE1 pathway may be cell-type dependent.

To address the referee's question, we have further examined the impact of mycolactone treatment, alone and combined with BZ, on activation of the PERK pathway in MM cells. Upon ER stress, PERK-mediated phosphorylation of eIF2α reprograms protein translation to promote the expression of stress response mRNAs such as ATF4 (*3*). ATF4 subsequently drives the expression of genes alleviating stress such as the growth arrest and DNA damage-inducible protein (GADD34), which promotes p-eIF2α dephosphorylation in a negative feedback loop. If cellular stress is unresolved, ATF4 promotes apoptosis through induction of C/EBP homology protein (CHOP) gene expression.

While we could not convincingly demonstrate enhanced phosphorylation of PERK and eIF2α by Western blot analyses of MM cell lysates (data not shown), we detected an increased expression of

p-eIF2α target ATF4 at both the mRNA and protein levels in MM.1S cells treated with the mycolactone/BZ combination, compared to single drug treatments. Elevated ATF4 correlated with enhanced expression of its CHOP target, reflecting the activation of the eIF2α/ATF4 signaling pathway. It was also associated with induction of ATF4 target GADD34, suggesting that dephosphorylation of p-eIF2α operates in treated MM.1S cells to downregulate this pathway. In addition to our previous quantification of ATF4 protein levels and CHOP gene transcripts in MM cells treated with the mycolactone/BZ combination or single drugs (Fig. 3C, D and Fig. EV2), we now show a qPCR analysis of ATF4 and GADD34 mRNA levels in treated MM.1S cells (Fig. 3B and D, respectively). Together, these data support the conclusion that the mycolactone/BZ drug combination is superior to single drugs in activation of eIF2α/ATF4/CHOP signaling in MM cells.

Referee #2

1. To target sec61 in MM cells, we must know the significance of sec61 translocon in MM cells, Desirée Schubert et al reported that SEC61A1-V85D triggered the terminal unfolded protein response in MM cells, and it disturbs the cellular calcium homeostasis in HeLa cells, thus they found it plays role in PC deficiency and pathogenesis of antibody deficiencies. What is the role and significance of sec61 in the clinic should be investigated.

We have not been able to conduct a genetic analysis of Sec61 in MM patient samples yet, due to regulatory constraints, but we plan to include Sec61 targeted sequencing in our future studies. To discuss the potential issues associated with Sec61 blockade in the clinic, our revised manuscript now includes an additional paragraph page 14, pasted below.

"Similar to PIs and IMiDs, the clinical use of Sec61 inhibitors raises questions about their potential toxicity in normal cells, and the risks to generate drug resistance in tumor cells. Mycolactone-mediated Sec61 blockade provokes perturbations in protein homeostasis whose magnitude and ability to evolve towards apoptosis vary across cell types, depending on their functional biology (*2*, *4*). Recent studies using computer simulations or lipid monolayers also suggested that mycolactone passage across cellular membranes may alter their integrity and dynamic properties (*5*, *6*). Using primary human cells, we reported previously that contrary to macrophages, neutrophils and T lymphocytes are resistant to mycolactone-mediated toxicity (*7*). The present study further indicates that MM cells are more susceptible to Sec61 blockade-driven apoptosis than PBMCs. In mice, repeated systemic administration of mycolactone significantly delayed MM growth without inducing toxic effects. Together, our *in vitro* and *in vivo* results thus define a therapeutic window for Sec61 blockers in MM treatment. Regarding the development of resistance in tumor cells, one can speculate that increased expression of Sec61α may reduce the inhibitor's ability to saturate Sec61 binding sites, thereby the induction of ER stress responses. Sec61α is a mycolactone-sensitive Sec61 client that was mildly upregulated by mycolactone in previous proteomic studies (*2*, *8*), suggesting that UPR-mediated increase in Sec61α gene expression can override Sec61 blockade, at least to a limited extent. Mutations in the Sec61α gene sequence resulting in loss of inhibitor binding, such as those introduced experimentally in cancer cell lines in previous research (*9*, *10*), may also confer MM tumors with resistance to Sec61 inhibitors. To our knowledge, none of these mutations has been reported in human clinical samples, suggesting that they may prevent the translocon from ensuring

essential functions. Yet, it will be important to verify if such genetic adaptations arise in MM cells subjected to Sec61 blocker pressure *in vitro* and in animal models of disease."

2. Abstract: Notably, this synergy was selective of cancer cells and extended to B cell acute lymphoblastic leukemia. Why the authors focus on MM but not on ALL?

We focused on MM because of its higher incidence and medical relevance, compared to ALL (https://www.cancer.net/). Our choice was also motivated by the fact that, due to its high secretory activity and reliance on the UPR to maintain protein homeostasis, MM provided us with a good system to evaluate the therapeutic potential of novel anti-cancer agents targeting ER stress.

3. Fig 1A, it seems mycolactone is more effective to JIM-3 and KMS-11 cells. Since these two cells are t(4;14) positive and express high level of methyltransferase NSD2, is it possible that mycolactone kills MM is NSD2 or H3K36me2 dependent?

By transducing HEK293 cells with the mycolactone-resistant R66G mutant of Sec61, we showed previously that mycolactone-driven cell apoptosis is strictly Sec61-dependent (*9*). Similar findings were obtained in *v-abl* cells (see Figure 1 below), confirming that Sec61 blockade is the molecular mechanism underpinning mycolactone cytotoxicity.

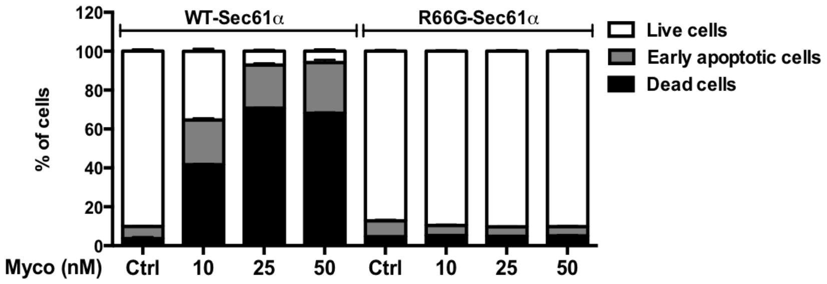

Figure 1: Mycolactone-driven apoptosis of *v-abl* cells is Sec61-dependent. Dose-dependent effects of mycolactone in *v-abl* cells transduced with wt- or R66G-Sec61α prior to 48h exposure to mycolactone. Annexin V+/PI- cells were identified as early apoptotic cells, Annexin V+/PI+- as late apoptotic cells, and Annexin V-/PI- cells as live cells. Data are mean percentages and are representative of two independent experiments using two *v-abl* clones.

Cytogenetic abnormalities are found in most MM patients, among which translocation t(4;14) that has been associated with poor prognosis (*11*). We thank Referee#2 for pointing out that mycolactone was more toxic in t(4;14) positive JIM3 and KMS-11 cells than in t(4;14) negative MM.1S cells. While a direct comparison between isogenic t(4;14) positive and negative cell lines would be required to conclude, this result indeed suggests that gene deregulation caused by NSD2 overexpression and expansion of H3K36me2 in t(4;14) MM cells (*12*) may confer them with enhanced susceptibility to the pro-death programs triggered by Sec61 blockade. This clinically relevant comment has been added to discussion page 14.

4. All three pathways of ER stress should be investigated.

As requested by Referee #1, the revised version of our manuscript now includes a more detailed analysis of mycolactone and BZ impact on the IRE1 and PERK pathways of ER stress. To complete this study, we also analyzed treatment effects on the third arm of the UPR, activated by the ER sensor ATF6, page 7:

“In MM.1S cells treated with BZ for 6 h, we observed defects in ATF6 glycosylation and partial cleavage of the protein (**Fig. 3E**). In agreement with previous reports (*1*), mycolactone did not induce such ATF6 cleavage. However, defective glycosylation and depletion of full length ATF6 were observed, likely resulting from mycolactone-mediated blockade of this Type II transmembrane protein in translocation. MM.1S cells exposed to the mycolactone-BZ combination displayed the sum of the alterations induced by each drug, namely depletion, defective glycosylation and partial cleavage of ATF6.”

5. Fig 6A, there is discrepancy in the figure legend and figure labels. Figure legend has 5 groups, but figure label only shows 4.

Corrected

Referee #3

The only additional aspect missing, to my mind, is comparison of mycolactone toxicity to that of common ER stress inducers, such as Tg (used as control in Figures 3-4), because presumably the relevant concentration would be considerably subtoxic when used alone and should induce much less ER stress than the Tg treatment.

In a previous study, we compared mycolactone to the common ER stressors thapsigargin, tunicamycin and MG132 for ability to activate the UPR in a dendritic cell line (*2*). Of all tested drugs, mycolactone was the less potent inducer of pro-apoptotic factor CHOP expression. To determine if this correlates with a relatively lower cytotoxicity, we performed a direct comparison of mycolactone and thapsigargin effects on viability of the MM.1S cell line:

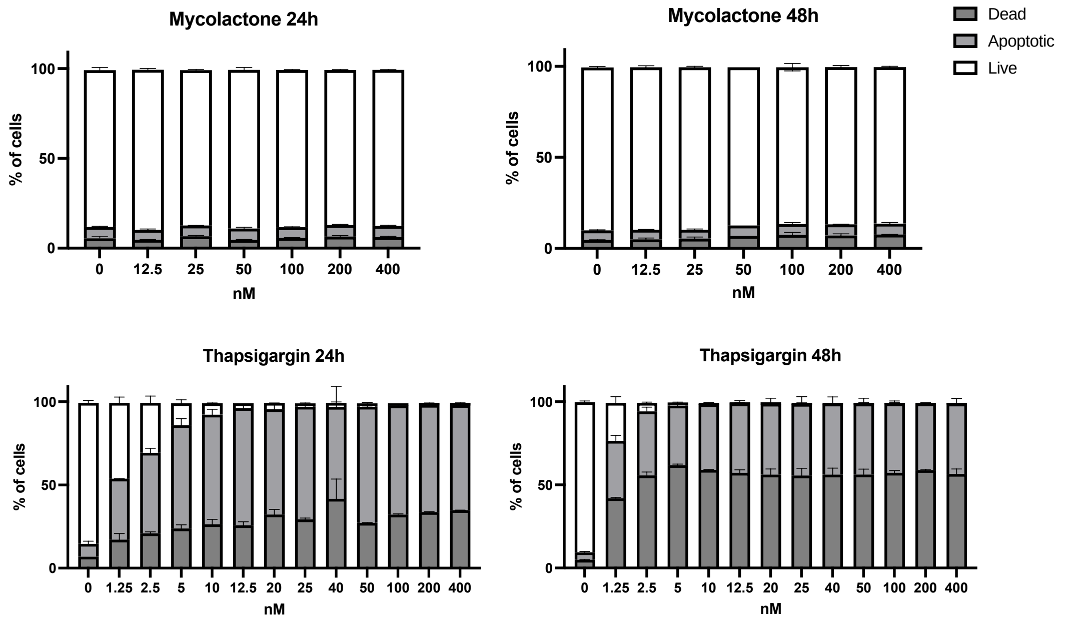

Figure 2: Compared cytotoxicities of mycolactone and thapsigargin in the MM.1S cell line. Effects of the two drugs, added at comparable concentrations, on the viability of MM.1S cells after 24h and 48h. Annexin V+/PI- cells were identified as early apoptotic cells, Annexin V+/PI+- as late apoptotic cells, and Annexin V-/PI- cells as live cells.

Confirming our data shown in Fig. 1A, these data show that mycolactone added at concentrations up to 400 nM did not alter MM.1S cell viability during the first 48h of treatment. In contrast, thapsigargin induced massive cell apoptosis after only 24h, at concentrations in the nanomolar range.

One shortcoming of the work is the absence of BZ-resistant cells that would represent the population which most critically would benefit from combination therapy. The work uses the KMS-11 line, which is relatively more resistant to BZ than the other cell lines (Fig. 2A), but among the patient-derived cells there are none that are models of BZ escape mutants. Importantly, the authors demonstrate the drug synergy in ex vivo patient cells (Figure 5) and show that the combination slows down tumor growth more than either BZ or mycolactone alone (Figure 6). I accept the conclusion but note that the relatively BZ-sensitive line MM.1s was used in this setup, so it remains to be seen if more resistant cells, or even BZ-refractive cells exhibit the same sensitivity.

To address this shortcoming and model the effect of Sec61 blockade on BZ escape mutants, we generated a MM.1S cell line with increased resistance to BZ, and compared its susceptibility to mycolactone, alone or combined to BZ, to that of the parental cell line. Data are reported in new Figure 4 and Result paragraph (page 8) pasted below :

"The cytotoxic synergy between mycolactone and BZ is maintained in BZ-resistant MM.1S cells

The development of resistance to PIs is a major obstacle to successful treatment of MM patients (*13*). To determine if the cytotoxic synergy between mycolactone and BZ was maintained in BZ-resistant MM cells, we generated a BZ-resistant version of the MM.1S cell line (MM.1S BzR cells), with stable and >15x increased resistance to BZ treatment (**Fig. 4A**). Notably, MM.1S BzR cell

susceptibility to mycolactone was identical to that of MM.1S BzS (**Fig. 4B and 1A**), showing that acquired resistance to BZ does not confer cross-resistance to mycolactone. As in MM.1S BzS cells (**Fig. 2C**), mycolactone synergized with BZ for induction of MM.1S BzR cell death (**Fig. 4C-D**) and engagement of apoptosis in MM.1S BzR cells exposed to the mycolactone-BZ combination correlated with activation of ER stress responses, reflected by elevated levels of ATF4, CHOP, and sXBP1 mRNAs (**Fig. 4E**). Like MM.1S BzS cells, MM.1S BzR cells failed to upregulate BiP expression upon exposure to BZ, mycolactone, or the drug combination (**Fig. 4E**). Together, these data demonstrate that mycolactone cytotoxicity and synergy with BZ override MM cell resistance to BZ. They consolidate the clinical relevance of Sec61 inhibitor-based treatments for MM."

An additional sentence has been added to discussion page 13, as follows:

"… mycolactone cytotoxicity and synergy with BZ were conserved in BZ-resistant MM cells. This indicates that mycolactone and BZ resistance mechanisms do not overlap and suggests that MM patients developing resistance to PIs will respond to Sec61 blockade therapy."

Figure S5: I find the layout of part B, the non-cancerous cells, somewhat misleading. The design of this figure is different from all the others, where the entire population is accounted for. For example, in the bottom panel, the viability in most conditions is less than 100, yet there are no details about cells that are not presented in the diagrams. This becomes important when one tries to interpret the effects of mycolactone on non-cancerous cells. The authors claim no effect on non-cancerous cells (p.8), even though the target is present in them, and this point is important for assessing the selectivity of the drug.

We have added an additional panel in ex Fig. 5B (now Fig. EV4B) to show the effect of drug treatments on viability of total PBMCs. Layout is comparable to that used in ex Fig. 5 (now Fig. 6), allowing direct comparisons between the effects of single and combined drug treatments on viability of patient-derived tumor cells (Fig. 6A) and PBMCs (Fig. EV4B), treated in the same conditions.

Additional questions

Possible mechanisms by which a drug to a ubiquitous target like sec61, which by itself has not cytotoxic effect, can synergize with another drug to kill MM cells more effectively,

In fact, mycolactone had a cytotoxic effect but it only manifested after 48h in MM cell lines (Fig. 1A and 4A) and *v-abl* cells (Fig. 5). In contrast, that of BZ manifested as soon as 24h in MM cell lines (Fig. 2 and 4). This is now better explained in the relevant paragraph of Results page 6. In experiments combining mycolactone and BZ, we looked at early time points (6h of drug exposure in analyses of ER stress, 24h of drug exposure in analyses of apoptosis) to capture synergistic effects. In all studied cells, the anti-MM activity of BZ was potentiated by co-incubation with mycolactone. To propose explanations for the synergy between mycolactone and BZ, we have performed analyses of the UPR regulator BiP in all cell lines. This ER-resident chaperone is normally induced by ER stress and

determines the threshold for induction of apoptosis. Results are shown in Figures 3, EV2 and 4, and they are discussed in an additional paragraph page 13 pasted below:

"Transition from adaptative to terminal UPR is primarily triggered by induction of ATF4/CHOP signaling (*3*), which was potently activated by the BZ-mycolactone combination in all studied MM cell lines. In plasma cells, high secretory activity associated with intracellular accumulation of misfolded immunoglobulin chains is believed to account for the limited ability of BiP to cope with PI-mediated ER stress and the particularly low threshold for induction of terminal UPR upon exposure to PIs in these cells (*14*). We reported previously that mycolactone-mediated UPR differs from that triggered by typical ER stressors by the lack of BiP induction (*9*). In line with this finding, despite displaying clear marks of ER stress MM cell lines failed to upregulate BiP expression upon exposure to mycolactone (**Fig. 3, Fig. EV2, Fig. 4**). In all cell lines, this defect was maintained in the presence of BZ. Therefore, we propose that mycolactone synergizes with BZ for induction of MM cell apoptosis by preventing BiP from regulating transition to terminal UPR."

Potential effects of chronic treatment with mycolactone in light of previous studies (i.e. PMID: 19934005 and 29320578)

These issues are now addressed in the additional discussion paragraph (see our answer to Referee #2, point 1).

References

1. J. Ogbechi, B. S. Hall, T. Sbarrato, J. Taunton, A. E. Willis, R. C. Wek, R. E. Simmonds, Inhibition of Sec61-dependent translocation by mycolactone uncouples the integrated stress response from ER stress, driving cytotoxicity via translational activation of ATF4. *Cell Death Dis*. **9**, 397 (2018).
2. J. D. Morel, A. O. Paatero, J. Wei, J. W. Yewdell, L. Guenin-Macé, D. Van Haver, F. Impens, N. Pietrosemoli, V. O. Paavilainen, C. Demangel, Proteomics reveals scope of mycolactone-mediated sec61 blockade and distinctive stress signature. *Mol. Cell. Proteomics*. **17**, 1750–1765 (2018).
3. C. Hetz, F. R. Papa, The Unfolded Protein Response and Cell Fate Control. *Mol. Cell* (2018), , doi:10.1016/j.molcel.2017.06.017.
4. C. Demangel, S. High, Sec61 blockade by mycolactone: a central mechanism in Buruli ulcer disease. *Biol. Cell* (2018), doi:10.1111/boc.201800030.
5. C. A. López, C. J. Unkefer, B. I. Swanson, J. M. J. Swanson, S. Gnanakaran, Membrane perturbing properties of toxin mycolactone from Mycobacterium ulcerans. *PLoS Comput. Biol.* **14**, e1005972 (2018).
6. M. Nitenberg, A. Benarouche, O. Maniti, E. Marion, L. Marsollier, J. Gean, E. J. Dufourc, J. F. Cavalier, S. Canaan, A. P. Girard-Egrot, The potent effect of mycolactone on lipid membranes. *PLoS Pathog*. **14**, e1006814 (2018).
7. L. Guenin-Mace, L. Baron, A. C. Chany, C. Tresse, S. Saint-Auret, F. Jonsson, F. Le Chevalier, P. Bruhns, G. Bismuth, S. Hidalgo-Lucas, J. F. Bisson, N. Blanchard, C. Demangel, Shaping mycolactone for therapeutic use against inflammatory disorders. *Sci Transl Med*. **7**, 289ra85

(2015).
8. J. E. Grotzke, P. Kozik, J. D. Morel, F. Impens, N. Pietrosemoli, P. Cresswell, S. Amigorena, C. Demangel, Sec61 blockade by mycolactone inhibits antigen cross-presentation independently of endosome-to-cytosol export. *Proc. Natl. Acad. Sci. U. S. A.* (2017), doi:10.1073/pnas.1705242114.
9. L. Baron, A. O. Paatero, J. D. Morel, F. Impens, L. Guenin-Macé, S. Saint-Auret, N. Blanchard, R. Dillmann, F. Niang, S. Pellegrini, J. Taunton, V. O. Paavilainen, C. Demangel, Mycolactone subverts immunity by selectively blocking the Sec61 translocon. *J. Exp. Med.* **213**, 2885–2896 (2016).
10. S. F. Gérard, B. S. Hall, A. M. Zaki, K. A. Corfield, P. U. Mayerhofer, C. Costa, D. K. Whelligan, P. C. Biggin, R. E. Simmonds, M. K. Higgins, Structure of the Inhibited State of the Sec Translocon. *Mol. Cell* (2020), doi:10.1016/j.molcel.2020.06.013.
11. N. Abdallah, S. V. Rajkumar, P. Greipp, P. Kapoor, M. A. Gertz, A. Dispenzieri, L. B. Baughn, M. Q. Lacy, S. R. Hayman, F. K. Buadi, D. Dingli, R. S. Go, Y. L. Hwa, A. Fonder, M. Hobbs, Y. Lin, N. Leung, T. Kourelis, R. Warsame, M. Siddiqui, J. Lust, R. A. Kyle, L. Bergsagel, R. Ketterling, S. K. Kumar, Cytogenetic abnormalities in multiple myeloma: association with disease characteristics and treatment response. *Blood Cancer J.* (2020), doi:10.1038/s41408-020-00348-5.
12. P. Lhoumaud, S. Badri, J. Rodriguez-Hernaez, T. Sakellaropoulos, G. Sethia, A. Kloetgen, M. I. Cornwell, S. Bhattacharyya, F. Ay, R. Bonneau, A. Tsirigos, J. A. Skok, NSD2 overexpression drives clustered chromatin and transcriptional changes in a subset of insulated domains. *Nat. Commun.* (2019), doi:10.1038/s41467-019-12811-4.
13. P. Lawasut, D. Chauhan, J. Laubach, C. Hayes, C. Fabre, M. Maglio, C. Mitsiades, T. Hideshima, K. C. Anderson, P. G. Richardson, New proteasome inhibitors in myeloma. *Curr. Hematol. Malig. Rep.* (2012), doi:10.1007/s11899-012-0141-2.
14. E. A. Obeng, L. M. Carlson, D. M. Gutman, W. J. Harrington Jr., K. P. Lee, L. H. Boise, Proteasome inhibitors induce a terminal unfolded protein response in multiple myeloma cells. *Blood*. **107**, 4907–4916 (2006).

2nd Dec 2021

Dear Caroline,

Thank you for the submission of your revised manuscript to EMBO Molecular Medicine. We have now received the enclosed reports from the three referees who re-reviewed your manuscript. As you will see, they are supportive of publication, and I am therefore pleased to inform you that we will be able to accept your manuscript, once the following minor points will be addressed:

1/ Please address the remaining minor concerns from referees #1 and #2.

2/ Main manuscript text
- Please remove the yellow highlights, and only keep in track changes mode any new modification.
- Material and methods:
o Patients: Please include the full statement that the experiments conformed to the principles set out in the WMA Declaration of Helsinki and the Department of Health and Human Services Belmont Report.
o Please indicate antibody dilutions/concentrations
- Thank you for providing a Data Availability section. In case you have no data that requires deposition in a public database, please indicate "This study includes no data deposited in external repositories". The rest of the text may be removed.

3/ Figures:
- Please indicate in the figures or in their legends the exact p= values, not a range. Some people found that to keep the figures clear, providing a supplemental table with all exact p-values was preferable. You are welcome to do this if you want to.
- Thank you for distinguishing between technical and biological replicates in the figure legends. We note that you have several figures that contain error bars based on n=2 (biological replicates). Please use scatter blots showing the individual datapoints in these cases. The use of statistical tests needs to be justified.

4/ Checklist:
B/ Statistics: please provide more information in section 5.
E/ Human subjects: please provide detailed information in sections 11 and 12.
F/ Data accessibility: please update section 18 (This study includes no data deposited in external repositories).

5/ Please note that all corresponding authors are required to supply an ORCID ID for their name upon submission of a revised manuscript. An ORCID is currently missing for G. Dadaglio.

6/ Every published paper now includes a 'Synopsis' to further enhance discoverability. Synopses are displayed on the journal webpage and are freely accessible to all readers. They include a short stand first (maximum of 300 characters, including space) as well as 2-5 one-sentences bullet points that summarizes the paper. Please write the bullet points to summarize the key NEW findings. They should be designed to be complementary to the abstract - i.e. not repeat the same text. We encourage inclusion of key acronyms and quantitative information (maximum of 30 words / bullet point). Please use the passive voice. Please attach these in a separate file or send them by email, we will incorporate them accordingly.

Please also suggest a striking image or visual abstract to illustrate your article as a PNG file 550 px wide x 300-600 px high.

7/ As part of the EMBO Publications transparent editorial process initiative (see our Editorial at http://embomolmed.embopress.org/content/2/9/329), EMBO Molecular Medicine will publish online a Review Process File (RPF) to accompany accepted manuscripts.
This file will be published in conjunction with your paper and will include the anonymous referee reports, your point-by-point response and all pertinent correspondence relating to the manuscript. Let us know whether you agree with the publication of the RPF and as here, IF YOU WANT TO REMOVE OR NOT any figures from it prior to publication.
Please note that the Authors checklist will be published at the end of the RPF.

I look forward to receiving your revised manuscript.

With kind regards,

Lise

Lise Roth, PhD

Editor
EMBO Molecular Medicine

To submit your manuscript, please follow this link:

Link Not Available

*Additional important information regarding Figures

Each figure should be given in a separate file and should have the following resolution:
Graphs 800-1,200 DPI
Photos 400-800 DPI
Colour (only CMYK) 300-400 DPI"

Figures are not edited by the production team. All lettering should be the same size and style; figure panels should be indicated by capital letters (A, B, C etc). Gridlines are not allowed except for log plots. Figures should be numbered in the order of their appearance in the text with Arabic numerals. Each Figure must have a separate legend and a caption is needed for each panel.

*Additional important information regarding figures and illustrations can be found at https://bit.ly/EMBOPressFigurePreparationGuideline. See also figure legend preparation guidelines: https://www.embopress.org/page/journal/17574684/authorguide#figureformat

The system will prompt you to fill in your funding and payment information. This will allow Wiley to send you a quote for the article processing charge (APC) in case of acceptance. This quote takes into account any reduction or fee waivers that you may be eligible for. Authors do not need to pay any fees before their manuscript is accepted and transferred to our publisher.

***** Reviewer's comments *****

Referee #1 (Remarks for Author):

The authors have made a heartfelt appeal to the reject decision. Their reply is based on experiments and new data, and their effort is palpable and commended. They now provide an exhaustive analysis of the UPR as suggested. The results are clearly presented and show activation of ATF4, lack of activation of GRP78 (per their previous report), little activation of ATF6, but surprisingly no phosphorylation of eIF2alpha even though there is a clear activation of ATF4/CHOP and GADD34.

This is perplexing. There are two potential explanations for this paradox. One, trivial they used a bad phospho antibody. The other is more complex is that mycolactone acts in a way similar to a new class of compounds (e.g., IXA4) that is a direct activator of XBP1 splicing without UPR (PMID: 32690944). This would explain their results but would still require an explanation as to how ATF4 is activated.

Therefore, while their effort to improve the manuscript is laudable, the data fall short of being explained. Based on this, I would refrain from stating " these data support the conclusion that the mycolactone/BZ drug combination is superior to single drugs in activation of eIF2alpha/ATF4/CHOP signaling in MM cells" because eIF2 phosphorylation this has not been proven. The authors should explain how this occurs. A possibility is that mycolactone keep the dephosphorylation active and as soon eIF2 is phosphorylated is also de-phosphorylated prevention translation inhibition and causing greater unresolvable UPR. Not it is clear what the sentence "mycolactone synergizes with BZ for induction of MM cell apoptosis by preventing BiP from regulating transition to terminal UPR" is supposed to mean. I would delete or explain better.
I realize the matter is complex, and the benefit of a new drug to MM patients may not need a full mechanistic explanation. The authors may add comments along the lines suggested in this review of their new work.

Referee #4 (Remarks for Author):

I appreciate all efforts that authors have done to answer my questions. I have only one suggestion:

Clinical significance including progression and clinical outcomes of sec61 in MM patients should be further confirmed using public database, such as GSE or coMMpass from MMRF.

Referee #5 (Comments on Novelty/Model System for Author):

The revised manuscript by Demangel et al is improved and I consider it suitable for publication in EMM.
In particular the authors answered satisfactorily my two main points about the first submission:
1. Bz resistant cell lines are now included in that analysis and show a similar synergism between mycolactone and proteasome inhibitors as the original cell lines. The synergism in a model of treatment resistant cell lines leads one to expect that the combination regime will be effective against ex vivo tumors.
2. A molecular mechanism is now proposed for the synergy - induction of a novel form of the unfolded protein response where the major chaperone BiP/GRP78 is not induced. This mechanism is feasible because it invokes a common factor that operates in the three known sensing mechanisms of accumulated unfolded proteins, the presumed trigger for the response in MM cells. Even though BiP has targets beyond the three UPR sensors, its lack of BiP induction would indeed be expected to lead to sensitization to apoptosis-induction.
3. My minor comments were also addressed in the revised manuscript.

The authors performed the requested editorial changes.

22nd Dec 2021

Dear Caroline,

Thank you for submitting your revised files. I am very pleased to inform you that your manuscript is now accepted for publication and will be sent to our publisher to be included in the next available issue of EMBO Molecular Medicine!

Please note that an ORCID identifier is still missing for G. Dadaglio. We realize that you provided the identifier in your cover letter, however only Dr. Dadaglio can link the identifier to his name, and we cannot do it for him.

We would like to remind you that as part of the EMBO Publications transparent editorial process initiative, EMBO Molecular Medicine will publish a Review Process File online to accompany accepted manuscripts. If you do NOT want the file to be published or would like to exclude figures, please immediately inform the editorial office via e-mail.

Please read below for additional IMPORTANT information regarding your article, its publication and the production process.

Congratulations on your interesting work!

With my best wishes for the holiday season,

Lise

Lise Roth, Ph.D
Scientific Editor
EMBO Molecular Medicine

EMBO PRESS

YOU MUST COMPLETE ALL CELLS WITH A PINK BACKGROUND

PLEASE NOTE THAT THIS CHECKLIST WILL BE PUBLISHED ALONGSIDE YOUR PAPER

Corresponding Author Name: DEMANGEL
Journal Submitted to: EMBO Molecular Medicine
Manuscript Number: EMM-2021-14740

Reporting Checklist For Life Sciences Articles (Rev. June 2017)

This checklist is used to ensure good reporting standards and to improve the reproducibility of published results. These guidelines are consistent with the Principles and Guidelines for Reporting Preclinical Research issued by the NIH in 2014. Please follow the journal's authorship guidelines in preparing your manuscript.

A- Figures
1. Data
The data shown in figures should satisfy the following conditions:

- the data were obtained and processed according to the field's best practice and are presented to reflect the results of the experiments in an accurate and unbiased manner.
- figure panels include only data points, measurements or observations that can be compared to each other in a scientifically meaningful way.
- graphs include clearly labeled error bars for independent experiments and sample sizes. Unless justified, error bars should not be shown for technical replicates.
- if n< 5, the individual data points from each experiment should be plotted and any statistical test employed should be justified
- Source Data should be included to report the data underlying graphs. Please follow the guidelines set out in the author ship guidelines on Data Presentation.

2. Captions
Each figure caption should contain the following information, for each panel where they are relevant:

- a specification of the experimental system investigated (eg cell line, species name).
- the assay(s) and method(s) used to carry out the reported observations and measurements
- an explicit mention of the biological and chemical entity(ies) that are being measured.
- an explicit mention of the biological and chemical entity(ies) that are altered/varied/perturbed in a controlled manner.

- the exact sample size (n) for each experimental group/condition, given as a number, not a range;
- a description of the sample collection allowing the reader to understand whether the samples represent technical or biological replicates (including how many animals, litters, cultures, etc.).
- a statement of how many times the experiment shown was independently replicated in the laboratory.
- definitions of statistical methods and measures:
  - common tests, such as t-test (please specify whether paired vs. unpaired), simple $\chi 2$ tests, Wilcoxon and Mann-Whitney tests, can be unambiguously identified by name only, but more complex techniques should be described in the methods section;
  - are tests one-sided or two-sided?
  - are there adjustments for multiple comparisons?
  - exact statistical test results, e.g., P values = x but not P values < x;
  - definition of 'center values' as median or average;
  - definition of error bars as s.d. or s.e.m.

Any descriptions too long for the figure legend should be included in the methods section and/or with the source data.

In the pink boxes below, please ensure that the answers to the following questions are reported in the manuscript itself. Every question should be answered. If the question is not relevant to your research, please write NA (non applicable). We encourage you to include a specific subsection in the methods section for statistics, reagents, animal models and human subjects.

USEFUL LINKS FOR COMPLETING THIS FORM

http://www.antibodypedia.com
http://1degreebio.org
http://www.equator-network.org/reporting-guidelines/improving-bioscience-research-repor
http://grants.nih.gov/grants/olaw/olaw.htm
http://www.mrc.ac.uk/Ourresearch/Ethicsresearchguidance/Useofanimals/index.htm
http://ClinicalTrials.gov
http://www.consort-statement.org
http://www.consort-statement.org/checklists/view/32-consort/66-title
http://www.equator-network.org/reporting-guidelines/reporting-recommendations-for-tumo
http://datadryad.org
http://figshare.com
http://www.ncbi.nlm.nih.gov/gap
http://www.ebi.ac.uk/ega
http://biomodels.net/
http://biomodels.net/miriam/
http://jjj.biochem.sun.ac.za
https://osp.od.nih.gov/biosafety-biosecurity-and-emerging-biotechnology/
http://www.selectagents.gov/

B- Statistics and general methods

Please fill out these boxes (Do not worry if you cannot see all your text once you press return)

1.a. How was the sample size chosen to ensure adequate power to detect a pre-specified effect size?	The exact number of replicates (technical and biological) is precised in figure legends. For each data experiment, a minimum of 2 technical replicates was performed and each cellular study was repeated at least twice independently.
1.b. For animal studies, include a statement about sample size estimate even if no statistical methods were used.	How sample size was determined in mouse studies is explained in legend of Figure 7.
2. Describe inclusion/exclusion criteria if samples or animals were excluded from the analysis. Were the criteria pre-established?	The following statement has been added to the relevant section of Materials and Methods : "No animal was excluded from our analyses."
3. Were any steps taken to minimize the effects of subjective bias when allocating animals/samples to treatment (e.g. randomization procedure)? If yes, please describe.	The following statement has been added to the relevant section of Materials and Methods : "After 24h, mice were randomly assigned to 4 groups and 6 days later, each group was randomly assigned a treatment."
For animal studies, include a statement about randomization even if no randomization was used.	The following statement has been added to the relevant section of Materials and Methods : "After 24h, mice were randomly assigned to 4 groups and 6 days later, each group was randomly assigned a treatment."
4.a. Were any steps taken to minimize the effects of subjective bias during group allocation or/and when assessing results (e.g. blinding of the investigator)? If yes please describe.	Yes for group allocation (see above), but not when assessing results (see below).
4.b. For animal studies, include a statement about blinding even if no blinding was done	The following statement has been added to the relevant section of Materials and Methods : "Tumor growth was assessed daily by unblinded measurement of tumor size with a digital caliper."
5. For every figure, are statistical tests justified as appropriate?	Detailed information on the statistical test used and number of replicates is provided in each figure legend. In our qPCR analyses, we performed nested one-way ANOVA to account for the nested structure of our experimental design that used technical replicates within biological replicates, and Tukey's test to account for multiple comparisons. In our mouse studies, difference between groups were analyzed with Tukey's multiple comparison test using Two-way Anova, with mixed effect model for each time points.
Do the data meet the assumptions of the tests (e.g., normal distribution)? Describe any methods used to assess it.	ANOVAs assume that residuals are sampled from Gaussian distributions, an assumption we considered reasonable after checking the dispersion of our data around means.

Is there an estimate of variation within each group of data?	In each qPCR experiment comparing groups, individual data points were plotted to show their spread around mean values.
Is the variance similar between the groups that are being statistically compared?	ANOVAs assume that residuals are sampled from Gaussian distributions, an assumption we considered reasonable after checking the dispersion of our data around means.

C- Reagents

6. To show that antibodies were profiled for use in the system under study (assay and species), provide a citation, catalog number and/or clone number, supplementary information or reference to an antibody validation profile. e.g., Antibodypedia (see link list at top right), 1DegreeBio (see link list at top right).	For each antibody used, provider and catalog number are provided in the relevant section of Materials and Methods.
7. Identify the source of cell lines and report if they were recently authenticated (e.g., by STR profiling) and tested for mycoplasma contamination.	The origin of cell lines is precised in the relevant section of Material and Methods. They were tested negative for mycoplasma upon reception in CD's lab, where all cellular studies were performed.

* for all hyperlinks, please see the table at the top right of the document

D- Animal Models

8. Report species, strain, gender, age of animals and genetic modification status where applicable. Please detail housing and husbandry conditions and the source of animals.	Mouse strain, gender and age are precised are provided in the relevant section of Materials and Methods.
9. For experiments involving live vertebrates, include a statement of compliance with ethical regulations and identify the committee(s) approving the experiments.	Compliance statement is provided are provided in the relevant section of Materials and Methods.
10. We recommend consulting the ARRIVE guidelines (see link list at top right) (PLoS Biol. 8(6), e1000412, 2010) to ensure that other relevant aspects of animal studies are adequately reported. See author guidelines, under 'Reporting Guidelines'. See also: NIH (see link list at top right) and MRC (see link list at top right) recommendations. Please confirm compliance.	I (CD) onfirm compliance.

E- Human Subjects

11. Identify the committee(s) approving the study protocol.	The protocol received approval of the ethical committee "Groupements hospitaliers universitaires" GHU Nord; N° 23798-2020012709469025 # 121.
12. Include a statement confirming that informed consent was obtained from all subjects and that the experiments conformed to the principles set out in the WMA Declaration of Helsinki and the Department of Health and Human Services Belmont Report.	This statement is provided in the "Patients" section of Materials and Methods.
13. For publication of patient photos, include a statement confirming that consent to publish was obtained.	NA
14. Report any restrictions on the availability (and/or on the use) of human data or samples.	NA
15. Report the clinical trial registration number (at ClinicalTrials.gov or equivalent), where applicable.	NA
16. For phase II and III randomized controlled trials, please refer to the CONSORT flow diagram (see link list at top right) and submit the CONSORT checklist (see link list at top right) with your submission. See author guidelines, under 'Reporting Guidelines'. Please confirm you have submitted this list.	NA
17. For tumor marker prognostic studies, we recommend that you follow the REMARK reporting guidelines (see link list at top right). See author guidelines, under 'Reporting Guidelines'. Please confirm you have followed these guidelines.	NA

F- Data Accessibility

18: Provide a "Data Availability" section at the end of the Materials & Methods, listing the accession codes for data generated in this study and deposited in a public database (e.g. RNA-Seq data: Gene Expression Omnibus GSE39462, Proteomics data: PRIDE PXD000208 etc.) Please refer to our author guidelines for 'Data Deposition'.<br><br>Data deposition in a public repository is mandatory for:<br>a. Protein, DNA and RNA sequences<br>b. Macromolecular structures<br>c. Crystallographic data for small molecules<br>d. Functional genomics data<br>e. Proteomics and molecular interactions	This study includes no data deposited in external repositories.
19. Deposition is strongly recommended for any datasets that are central and integral to the study; please consider the journal's data policy. If no structured public repository exists for a given data type, we encourage the provision of datasets in the manuscript as a Supplementary Document (see author guidelines under 'Expanded View' or in unstructured repositories such as Dryad (see link list at top right) or Figshare (see link list at top right).	NA
20. Access to human clinical and genomic datasets should be provided with as few restrictions as possible while respecting ethical obligations to the patients and relevant medical and legal issues. If practically possible and compatible with the individual consent agreement used in the study, such data should be deposited in one of the major public access-controlled repositories such as dbGAP (see link list at top right) or EGA (see link list at top right).	NA
21. Computational models that are central and integral to a study should be shared without restrictions and provided in a machine-readable form. The relevant accession numbers or links should be provided. When possible, standardized format (SBML, CellML) should be used instead of scripts (e.g. MATLAB). Authors are strongly encouraged to follow the MIRIAM guidelines (see link list at top right) and deposit their model in a public database such as Biomodels (see link list at top right) or JWS Online (see link list at top right). If computer source code is provided with the paper, it should be deposited in a public repository or included in supplementary information.	NA

G- Dual use research of concern

22. Could your study fall under dual use research restrictions? Please check biosecurity documents (see link list at top right) and list of select agents and toxins (APHIS/CDC) (see link list at top right). According to our biosecurity guidelines, provide a statement only if it could.	NA
